# Fluorinated Organic Cations Derived Chiral 2D Perovskite Enabling Enhanced Spin‐Dependent Oxygen Evolution Reaction

**DOI:** 10.1002/advs.202403326

**Published:** 2024-06-28

**Authors:** Jaehyun Son, Gyumin Jang, Sunihl Ma, Hyungsoo Lee, Chan Uk Lee, Seongyeon Yang, Junwoo Lee, Subin Moon, Wooyong Jeong, Jeong Hyun Park, Chan‐Woo Jung, Ji‐Hee Kim, Ji‐Sang Park, Jooho Moon

**Affiliations:** ^1^ Department of Materials Science and Engineering Yonsei University Seoul 03722 Republic of Korea; ^2^ Department of Chemical Engineering University of Michigan Ann Arbor MI 48109 USA; ^3^ Department of Energy Science Sungkyunkwan University Suwon 16419 Republic of Korea; ^4^ Department of Physics Pusan National University Busan 46241 Republic of Korea; ^5^ Department of Nano Engineering Sungkyunkwan University Suwon 16419 Republic of Korea

**Keywords:** cation engineering, chiral perovskite, chirality‐induced spin selectivity, spin‐dependent oxygen evolution reaction, water stability

## Abstract

Chirality‐induced spin selectivity observed in chiral 2D organic–inorganic hybrid perovskite holds promise to achieve spin‐dependent electrochemistry. However, conventional chiral 2D perovskites suffer from low conductivity and hygroscopicity, limiting electrochemical performance and operational stability. Here, a cutting‐edge material design is introduced to develop a stable and efficient chiral perovskite‐based spin polarizer by employing fluorinated chiral cation. The fluorination approach effectively promotes the charge carrier transport along the out‐of‐plane direction by mitigating the dielectric confinement effect within the multi‐quantum well‐structured 2D perovskite. Integrating the fluorinated cation incorporated spin polarizer with BiVO_4_ photoanode considerably boosts the photocurrent density while reducing overpotential through a spin‐dependent oxygen evolution reaction. Furthermore, the hydrophobic nature of fluorine in spin polarizer endows operational stability to the photoanode, extending the durability by 280% as compared to the device with non‐fluorinated spin polarizer.

## Graphical abstract

The fluorinated chiral cation effectively reduces the exciton binding energy of the chiral organic‐inorganic 2D hybrid perovskite, leading to higher conductivity with excellent spin polarization. The photoelectrochemical device based on the fluorinated chiral perovskite achieves a 37% increase in photocurrent density and a 280% extension in operational stability during the spin‐dependent oxygen evolution reaction compared to the non‐fluorinated counterpart.

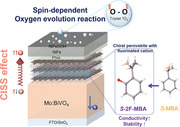

## 1 Introduction

Sluggish oxygen evolution reaction (OER) during photoelectrochemical (PEC) water splitting results in significant energy loss due to complex four‐electron transfer reactions.^[^ ^1^ ^]^ Mitigating the OER kinetic barrier at the photoanode is imperative to enhance overall solar‐to‐hydrogen efficiency.^[^ ^2^, ^3^ ^]^ Recently, spin‐dependent electrochemistry (SDE) offers an innovative approach to modulate multi‐step OER reactions by aligning the spin state of the charge carriers.^[^ ^4^ ^]^ Theoretically, spin‐polarized carriers promote triplet oxygen (^3^O_2_) generation while inhibiting energetically unfavored singlet oxygen (^1^O_2_) formation and the competitive by‐product, hydrogen peroxide (H_2_O_2_).^[^ ^5^, ^6^, ^7^ ^]^ SDE generally necessitates an external magnetic field to accomplish carrier spin‐polarization at room temperature.^[^ ^8^, ^9^ ^]^ In contrast, chiral materials, characterized by their non‐superimposable mirror images, have gained notable attention in the field of SDE due to their impressive spin‐polarizability without a need for external magnets.^[^ ^10^, ^11^, ^12^ ^]^ Specifically, the helical potential in the chiral materials, which inherently generates an effective magnetic field, can effectively polarize the spin states of charge carriers, known as chirality‐induced spin‐selectivity (CISS).^[^ ^13^ ^]^

To date, the CISS phenomenon observed in chiral materials, such as organic molecular monolayers^[^ ^14^, ^15^, ^16^ ^]^ and chiral inorganic compounds,^[^ ^6^, ^12^, ^17^, ^18^ ^]^ has been applied to address the sluggish kinetics during OER. However, such chiral materials suffer from a low degree of spin polarization (*P*_spin_), resulting in limited spin state manipulation. Recently, multi‐layered chiral organic–inorganic hybrid 2D perovskite (cOIHP) has been proposed as an ideal spin polarizer owing to the synergetic effect between significant chirality of the organic molecules and large spin‐orbital coupling of the inorganic layers.^[^ ^13^, ^19^ ^]^ The chiral organic cations, sandwiched between the inorganic layers in cOIHP, are able to generate repetitive helical potentials that align the spin state of the transporting charge carriers through multiple CISS processes, leading to a high degree of *P*_spin_ exceeding 80%.^[^ ^20^, ^21^, ^22^ ^]^ Furthermore, the cOIHP is solution‐processible, enabling facile heterostructure formation with conventional semiconductors. Based on these advantages, the SDE‐derived photoanode device with embedded cOIHP on Mo‐doped BiVO_4_ (BVO) demonstrated enhanced OER kinetics compared to photoanode without CISS effect.^[^ ^23^ ^]^

Despite the promising spin polarization capability of cOIHP‐embedded photoanode, its low conductivity poses inherent challenges that impede further improvement in PEC performance. Poor conductivity mainly stems from the multi‐quantum well (MQW) structured cOIHP, causing the dielectric confinement effect between the low dielectric constant of the chiral organic layer (ε_organic_) and the high dielectric constant of the inorganic layer (ε_inorganic_).^[^ ^24^, ^25^, ^26^ ^]^ The significant dielectric constant mismatch leads to huge exciton binding energy (E_b_), which confines carriers within MQW,^[^ ^27^ ^]^ consequently impairing out‐of‐plane conductivity where the CISS phenomenon develops.^[^ ^19^ ^]^ Meanwhile, the water instability of cOIHP is another hindrance to the spin‐dependent OER on the photoanode. The hygroscopic nature of the organic molecules makes cOIHP vulnerable to water, hampering long‐term operational stability.^[^ ^28^, ^29^ ^]^ Therefore, it is necessary to develop a rational material design to simultaneously modulate the out‐of‐plane conductivity and the hydrophobicity of cOIHP for improved spin‐dependent OER performance while guaranteeing durability.

Herein, we present a novel cOIHP spin polarizer through fluorination of the chiral cation, changing from conventional (*S*)‐methyl‐benzylamine (*S*‐MBA) to (*S*)‐ortho‐fluorinated‐methyl‐benzylamine (*S‐*2F‐MBA). *S*‐2F‐MBA cation incorporated within cOIHP (hereafter, *S*‐2F‐MBA cOIHP) is proficient in mitigating the dielectric confinement effect because the most electronegative fluorine atom can remarkably increase ε_organic_.^[^ ^30^, ^31^ ^]^ As a result, the diminished E_b_ in *S‐*2F‐MBA cOIHP significantly facilitates spin‐polarized charge transport through the out‐of‐plane direction compared to the *S*‐MBA‐based cOIHP (hereafter, S‐MBA cOIHP). We integrate *S‐*2F‐MBA cOIHP into the BVO photoanode as an embedded spin polarizer (denoted as BVO_*S*‐2F‐MBA). The resulting BVO_*S*‐2F‐MBA device can more effectively transfer spin‐polarized holes to the catalytic surface when compared to *S*‐MBA cOIHP‐embedded device (denoted as BVO_*S*‐MBA). Consequently, the BVO_*S*‐2F‐MBA exhibits a significantly higher photocurrent density of 4.6 mA cm⁻^2^ for OER with respect to the *S*‐MBA_BVO counterpart (3.35 mA cm⁻^2^), while suppressing by‐product H_2_O_2_ formation. Moreover, the hydrophobic characteristic of fluorine reinforces the water stability^[^ ^31^ ^]^ of the *S*‐2F‐MBA cOIHP spin polarizer, along with the synergetic effect with specific device architecture design, dramatically improving operational stability up to 6 h in a polar electrolyte without any encapsulation. Our findings suggest a promising design rationale for a highly conductive and stable cOIHP spin polarizer to implement the enhanced spin‐dependent OER on the photoanode.

## 2 Results and Discussion

### 2.1 Fabrication of Spin‐Dependent OER Photoanode by Integrating cOIHP on BVO

**Figure 1:**
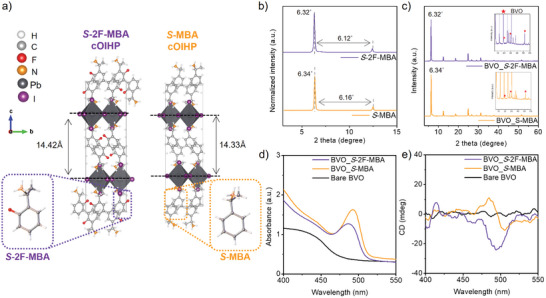
a) Molecular configuration of *S*‐2F‐MBA and *S*‐MBA cations as well as the crystal structure of the (*S*‐2F‐MBA)_2_PbI_4_ and (*S*‐MBA)_2_PbI_4_ cOIHP, respectively. The structure of the *S*‐MBA cOIHP crystal was redrawn based on the Cambridge Crystallographic Data Centre (CCDC) number 2015617.^[^ ^32^ ^]^ b) XRD patterns of the pristine cOIHP thin‐films. c) XRD patterns of the BVO_cOIHP structures. The insets are zoomed‐in XRD patterns for identifying the BVO peaks as represented by red stars. d) CD spectra and e) absorbance spectra obtained from the BVO_cOIHP structures.

**Figure** **1**a visualizes the crystal structures of (*S*‐2F‐MBA)_2_PbI_4_ and (*S*‐MBA)_2_PbI_4_ cOIHPs incorporating *S‐*2F‐MBA (see Table S1, Supporting Information for detailed structural parameters) and *S*‐MBA cations,^[^ ^32^ ^]^ respectively. Both chiral cations crystallized into Ruddlesden‐Popper 2D layered structure, where they are sandwiched between (PbI_6_)^4‐^ inorganic octahedra. We found that the *S*‐2F‐MBA cOIHP belongs to the globally centrosymmetric P_21_/n space group. However, it is worth noting that the local inversion symmetry breaking in the inorganic sublattice should be regarded as a crucial structural descriptor for identifying chirality in 2D organic‐inorganic perovskite structures beyond the global space group, emphasizing the necessity to consider local structural details in determining the chirality of cOIHPs. Therefore, we compare the inorganic layer distortion angles of the *S*‐2F‐MBA cOIHP with the racemic (*rac*)−2F‐MBA cOIHP, which also belongs to the centrosymmetric $P\bar{1}$ space group as shown in Figure S1 (Supporting Information). Remarkably, the inversion symmetry was broken in the inorganic framework of the *S*‐2F‐MBA cOIHP, whereas the inversion centers are retained at the Pb atoms of the *rac*‐2F‐MBA OIHP inorganic frameworks (i.e., the same angle exists on the opposite side). In other words, although both *S*‐2F‐MBA cOIHP and *rac*‐2F‐MBA cOIHP are assigned to the globally centrosymmetric space group, only the *S*‐2F‐MBA cOIHP reveals a broken inversion symmetry feature in the inorganic framework. Therefore, these observations validate that the chirality of the *S*‐2F‐MBA cation is structurally transferred into the inorganic framework by breaking the inversion symmetry of the inorganic framework.^[^ ^32^, ^33^ ^]^ Subsequently, X‐ray diffraction (XRD) spectra obtained from cOIHP thin films showed repeated sharp diffraction peaks of the (002*l*) plane at regular intervals (Figure 1b), indicating both layered cOIHP structures were parallelly grown on the fluorine‐doped tin oxide (FTO) glass substrate.^[^ ^21^ ^]^ The lowest 2θ peak, which indicates the (002) plane, of the S‐2F‐MBA cOIHP thin film at 6.32° was slightly shifted to smaller degrees from 6.34° of the *S*‐MBA cOIHP due to the steric effect of fluorine (Figure 1a). Notably, when the cOIHP layer was deposited on the BVO surface, the XRD spectra showed that both layered cOIHPs were successfully grown in parallel on top of the BVO (Figure 1c), as illustrated in Figure S2a (Supporting Information). The scanning electron microscope (SEM) images demonstrate that the nanoporous surface of bare BVO (*i.e*, without cOIHP) was entirely covered by the 80 nm‐thick cOIHP with smooth surface morphology (Figure S2b–e, Supporting Information). Furthermore, the BVO_cOIHP structure showed distinct circular dichroism (CD) spectra ≈490 nm (Figure 1d), unlike bare BVO. This CD signal originates from the intrinsic chirality of the cOIHPs,^[^ ^22^, ^34^ ^]^ as confirmed by the CD of the pristine cOIHP thin films (Figure S3a, Supporting Information). Moreover, the absorbance spectra of the BVO_cOIHP structure further evidence that the layered structure and chirality of the cOIHP were maintained in the heterostructure with BVO (Figure 1e; Figure S3b, Supporting Information). Because the optical interference possibly originates from linear birefringence and linear dichroism (LDLB) effect, the transmittance CD measurement of the solid‐state thin film may contaminate the observed CD spectra.^[^ ^35^, ^36^, ^37^ ^]^ In this regard, the genuine CD (CD_true_) of the cOIHP was evaluated by flipping the BVO_cOIHP samples according to the light propagation axis (Figure S4a,b, Supporting Information), revealing that the calculated CD_true_ is marginally affected by optical interference. Moreover, the anisotropy factor of the CD_true_ in Figure S4c (Supporting Information) revealed that both BVO_*S*‐2F‐MBA and BVO_*S*‐MBA possess the same handedness owing to the identical (*S*)‐ configuration of the chiral cation incorporated in cOHIP structure.

**Figure 2:**
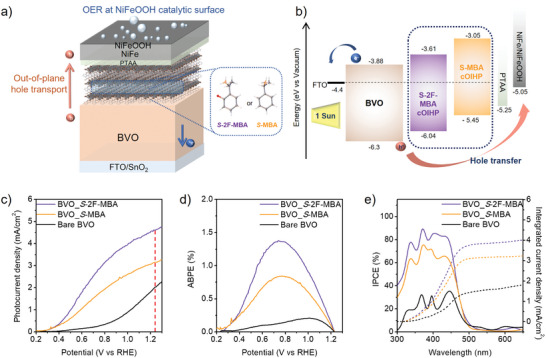
a) Schematic illustration of the fabricated photoanode device with the embedded spin polarizer. b) Schematic equilibrium energy band diagram of the photoanode device. The energy level information of the BVO and S‐MBA cOIHP was referred from the previously reported study.^[^ ^23^ ^]^ c) LSV curve for bare BVO and BVO_cOIHP devices measured in a 1 m potassium borate (K‐Bi) solution (pH 9.0) under continuous AM 1.5G back illumination. d) ABPE calculated from the LSV curves. e) IPCE spectra measured at 1.23 V_RHE_ under monochromatic light irradiation. The solid line represents IPCE and the dotted line indicates the integrated current density.

**Figure** **2**a depicts the photoanode configuration of FTO/SnO_2_/BVO/cOIHP/poly[bis(4‐phenyl)(2,4,6‐trimethylphenyl)amine](PTAA)/NiFe/NiFeOOH for OER analysis. The non‐polar PTAA was selected as a suitable hole transport layer (HTL) in the OER photoanode device.^[^ ^38^ ^]^ Moreover, the NiFe (≈16 nm) served as a physical barrier, preventing the water‐sensitive cOIHP layer from direct exposure to the polar solvent. It is worth noting that this cOIHP‐buried device architecture differs from the previously presented spin‐dependent OER photoanode based on the cOIHP^[^ ^23^ ^]^ in that ultrathin TiO_2_ protecting layer was replaced with NiFe ferromagnetic layer, to effectively preserve the spin state of the charge carriers. Furthermore, the catalytic layer has been changed from the particle‐transferred NiFeO_x_ to the electrodeposited highly active NiFeOOH (see details in the Experimental Section). Importantly, to scrutinize carrier transfer behavior for OER, the overall equilibrium band diagram of the photoanode device (Figure 2b) was determined using ultraviolet photoelectron spectroscopy analysis (Figure S5, Supporting Information).^[^ ^21^, ^23^ ^]^ Figure 2b demonstrated that photogenerated holes developed within the BVO photoabsorber can spontaneously transfer to the NiFeOOH catalytic layer, passing through the cOIHP spin polarizer, thereby inducing spin‐dependent OER at the catalytic surface. To verify the energy band structure, photoluminescence (PL) analyses were conducted using BVO_cOIHP configuration to confirm the hole transport at the BVO and cOIHP heterojunction (Figure S6a, Supporting Information). Importantly, the bare BVO exhibited a broad PL peak centered near 512 nm, whereas the PL peak of BVO_cOIHP structure was red‐shifted to 530 nm, which corresponds to the peak position of the pristine cOIHP thin film (Figure S6b, Supporting Information). Such a bathochromic PL shift indicated that photoexcited holes in the BVO photoabsorber are effectively transferred to cOIHP spin polarizer (Figure 2b).^[^ ^39^ ^]^ Therefore, the band diagram confirmed that our modified photoanode architecture effectively works for the spin‐dependent OER at the catalytic surface while promoting stable operation without the need for additional encapsulation.

Subsequently, linear sweep voltammetry (LSV) analyses were conducted with each device in a K‐Bi electrolyte (pH 9) under back illumination (Figure 2c). The control sample of a bare BVO device (i.e., without spin polarizer) exhibited a photocurrent density of 1.96 mA cm⁻^2^ at 1.23 V versus the reversible hydrogen electrode (V_RHE_) with an onset potential (V_onset_) of ≈0.8 V_RHE_. Surprisingly, a substantially improved OER performance was observed upon the introduction of cOIHP on the BVO. Particularly, the BVO_*S*‐2F‐MBA and BVO_*S*‐MBA devices delivered the best photocurrent density of 4.6 and 3.35 mA cm⁻^2^ (at 1.23 V_RHE_), respectively, with significantly low V_onset_ (< 0.4 V_RHE_). Remarkably, the simultaneous enhancements in photocurrent density and V_onset_ as well as fill factor led to a notable increase in the applied bias photon‐to‐current conversion efficiency (ABPE). As shown in Figure 2d, the ABPE for bare BVO was 0.21% (at 1.01 V_RHE_), which increased to 0.85% (at 0.77 V_RHE_) for BVO_*S*‐MBA and 1.39% (at 0.73 V_RHE_) for BVO_*S*‐2F‐MBA. Moreover, the incident‐photon‐to‐current‐efficiency (IPCE) also confirmed that the photoanode devices with cOIHP exhibited higher IPCE compared to bare BVO across the entire spectral region (Figure 2d), and the integrated photocurrent density is well matched with the LSV curve in Figure 2c. To unravel the origin of the significantly enhanced PEC performance upon integrating the cOIHP, it is essential to explore whether the cOIHP affects catalytic activity or contributes to the production of additional photoexcited carriers. Therefore, we compared LSV results of the BVO_cOIHP and cOIHP‐only OER devices (i.e., without BVO, Figure S7, Supporting Information) under either illumination or darkness, revealing that the presence of the cOIHP layer has a negligible effect on the electrochemical behavior of the NiFeOOH catalyst under dark condition. Moreover, in contrast to the superior light‐harvesting behavior observed in the BVO_cOHIP devices (Figure S7a, Supporting Information), the cOIHP‐only devices generated an infinitesimal photocurrent density under illumination condition (≈0.1 mA cm⁻^1^, Figure S7b, Supporting Information). This suggested that the photoelectric conversion efficiency from cOIHP itself was extremely low, primarily due to low dimension,^[^ ^40^ ^]^ confirming no extra photogeneration by the embedded cOHIP.

### 2.2 CISS Effect of the cOIHP Spin Polarizers

**Figure 3:**
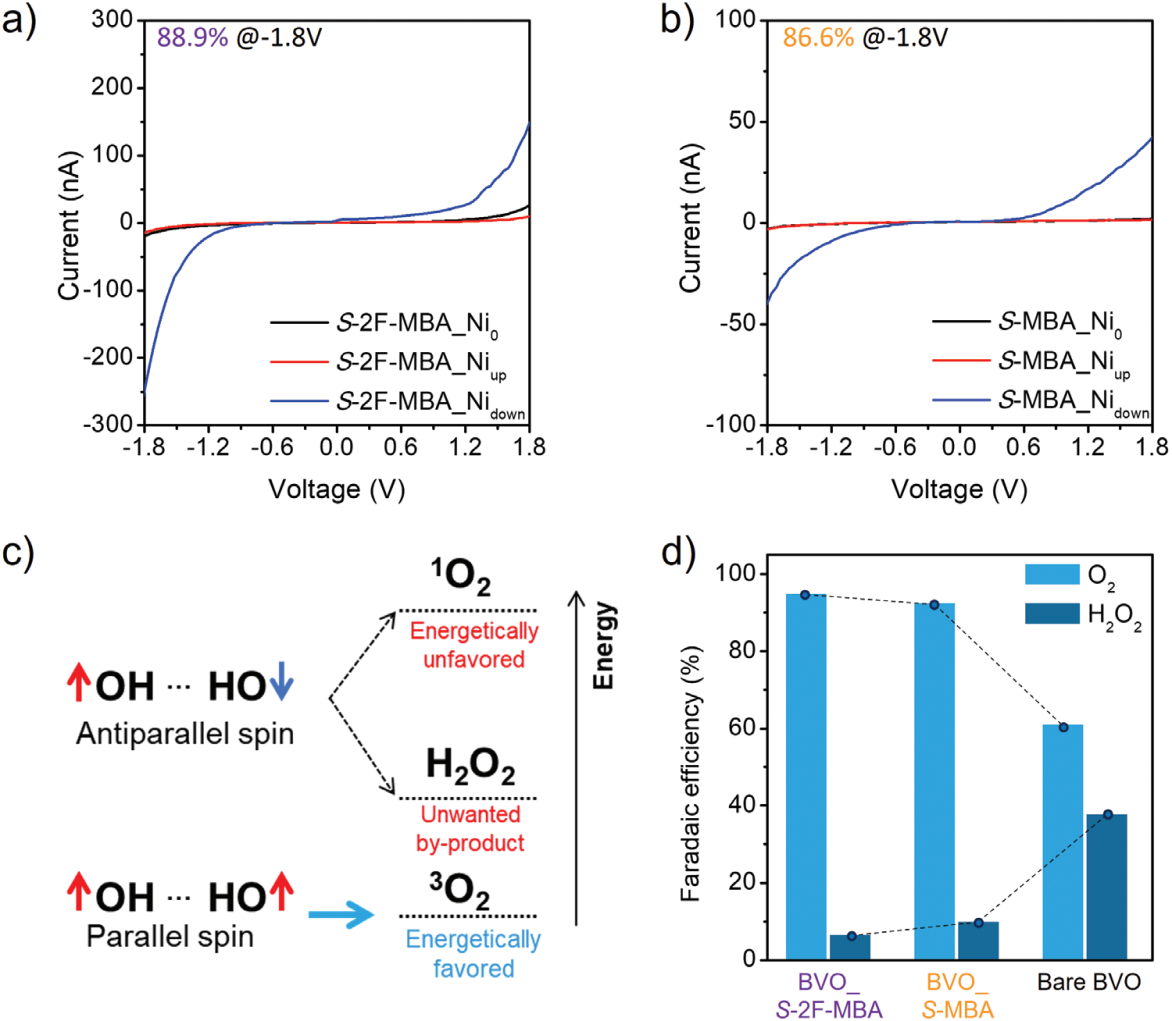
The *I*‐*V* curves of the CISS measurement devices averaged from 10 times measurements for each Ni magnetization direction for a) *S*‐2F‐MBA cOIHP and b) S‐MBA cOIHP. The black, blue, and red lines represent the Ni pre‐magnetization directions of non‐magnetization (Ni_0_), down (Ni_down_), and up (Ni_up_) direction, respectively. c) Theoretical energy diagram of spin‐dependent OER mechanism. The spin‐aligned OH^•^ radicals only allow the formation of triplet oxygen ^3^O_2_, while OH^•^ radicals with antiparallel spin states produce energetically unfavored singlet oxygen ^1^O_2_ as well as unwanted by‐product of H_2_O_2_. d) Calculated O_2_ and H_2_O_2_ Faradic efficiency stacked bar chart of the BVO_*S*‐2F‐MBA, BVO_*S*‐MBA, and bare BVO devices.

We also fabricated the BVO photoanode with racemic (*rac*)‐OIHPs, incorporating *rac*‐MBA and *rac*‐2F‐MBA cations respectively, and verified the absence of the chirality within both rac‐MBA OIHP and rac‐2F‐MBA OIHP using CD spectra (Figure S8, Supporting Information). Notably the BVO_*rac*‐OIHP device exhibited relatively inferior PEC performance, as characterized by lower photocurrent density and higher V_onset_ (refer to LSV curve in Figure S9, Supporting Information) compared to the BVO_cOIHP devices (Figure 2c). Therefore, it is reasonably speculated that the simultaneous improvement in photocurrent density and V_onset_ in BVO_cOIHP devices likely arises from the facilitated transfer of spin‐controlled photogenerated holes from BVO to the NiFeOOH catalyst by passing through cOIHP spin polarizer.^[^ ^18^, ^19^, ^20^, ^21^, ^22^ ^]^ In this context, the spin‐dependent carrier dynamics of cOIHP with different cations were verified by confirming the CISS effect using the properly fabricated devices (hereafter, CISS measurement devices), following the previously reported protocol.^[^ ^41^, ^42^ ^]^ As shown in Figure S10 (Supporting Information), the cOIHP layer was deposited on the Ni/Al_2_O_3_ CISS measurement device substrate with a thickness of 80 nm, which is comparable to the cOIHP spin polarizer on the BVO (Figure S1e, Supporting Information). The ferromagnetic Ni electrode at the bottom can polarize the spins of injected carriers into a specific state depending on the pre‐magnetization direction. Therefore, the spin‐polarized carriers injected from Ni layers travel through cOIHP, where multiple CISS processes occur in the out‐of‐plane direction. In this case, the degree of spin‐polarized current (*P*_spin_) can be calculated to quantitatively determine the CISS effect based on Equation (1):

(1) $$P_{\mathrm{spin}}=\;\frac{I_{\mathrm{up}\;}-\;I_{\mathrm{down}}}{I_{\mathrm{up}}\;+\;I_{\mathrm{down}}}\;\times \;100\%$$

where *I*_up_ and *I*_down_ represent the averaged current value from 10 times measurements as a function of up‐ or down‐magnetization directions, respectively. The results of the CISS measurement are shown in **Figure** **3**a,b, along with the raw data plotted in Figure S11 (Supporting Information). Remarkably, the CISS measurement devices fabricated with both *S*‐2F‐MBA and *S‐*MBA cOIHPs showed a higher *I*_down_ compared to *I*_up_, having *P*_spin_ of 88.9% for *S‐*2F‐MBA cOIHP (*I*_down_ of −249 nA and *I*_up_ of −14.6 nA at −1.8 V) and *P*_spin_ of 86.6% for *S‐*MBA cOIHP (*I*_down_ of −39.6 nA and *I*_up_ of −2.84 nA at −1.8 V).^[^ ^41^ ^]^ These results indicated that both cOIHP act as spin polarizers along the out‐of‐plane direction relative to the substrate, preferring the down‐spin state because of the same chirality (Figure S4, Supporting Information). In contrast, both *rac*‐2F‐MBA and *rac*‐MBA OIHPs lack spin polarizability in which the *I*_up_ and *I*_down_ were nearly identical in CISS measurement (Figure S12, Supporting Information). Although the photocurrent of the BVO_*rac*‐OIHP device revealed slight improvement compared to the bare BVO (Figure S9, Supporting Information), furthermore, its V_onset_ of ≈0.5 V_RHE_ is significantly higher than that of the BVO_cOIHP device (< 0.4 V_RHE_, Figure 2c). Given that high V_onset_ is primarily attributed to the sluggish OER kinetic barrier,^[^ ^10^, ^43^, ^44^ ^]^ the inferior OER performance of the BVO_*rac*‐OIHP (Figure S7, Supporting Information), as manifested by higher V_onset_, lower photocurrent density, and poor fill factor relative to the BVO_cOIHP (Figure 2c), underscores the importance of exploring the spin‐dependent OER kinetics upon the embedment of cOIHP.

### 2.3 Elucidation of the Spin‐Dependent OER Mechanism

Fundamentally, the OER involving a complex four‐electron transfer faces significant kinetic barriers arising from extensive side reactions during the OH^•^ intermediates formation.^[^ ^6^ ^]^ However, SDE can quantum mechanically facilitate the OER kinetics by restricting these sluggish reaction paths. As illustrated in the energy diagram in Figure 3c, when two OH^•^ radicals have parallel spin states, the energetically favorable ^3^O_2_ generation becomes predominant, while the formations of unfavored ^1^O_2_ and H_2_O_2_ by‐product are spin‐forbidden.^[^ ^2^, ^5^, ^10^, ^14^ ^]^ In this context, the photogenerated holes from BVO undergo spin polarization as they transit through cOIHP layer, achieving parallel spin orientation at the NiFeOOH catalytic surface (Figure 2b).^[^ ^19^, ^41^ ^]^ Therefore, the formation of sluggish products (i.e., ^1^O_2_ and H_2_O_2_) is diminished, thereby accelerating OER kinetics.^[^ ^7^, ^18^ ^]^ Conversely, in the absence of cOIHP spin polarizer (i.e., bare BVO and BVO_*rac*‐OIHP devices), the spin states of the OH^•^ radicals would be anti‐parallel with each other due to the Pauli exclusion principle. To verify whether the CISS effect‐derived spin‐dependent OER mechanism indeed suppresses the sluggish reaction (i.e., the formation of H_2_O_2_), we performed the colorimetric analysis using *o*‐tolidine.^[^ ^11^, ^17^, ^45^ ^]^ The amounts of H_2_O_2_ were measured using the absorbance peak intensity at 437 nm with UV–vis spectroscopy (Figure S13, Supporting Information). As shown in Figure S14 (Supporting Information), after 60 min of oxygen evolution, the BVO_cOIHP device generated only a minimal amount of H_2_O_2_, suggesting that spin‐dependent OER effectively hampers H_2_O_2_ formation. Conversely, the H_2_O_2_ formation over bare BVO and BVO_*rac*‐OIHP devices was noticeably increased, indicating that the sluggish reaction paths are allowed due to the absence of the CISS effect (Figure 3c). Furthermore, the Faradaic efficiencies of the production of the O_2_ (FE_O2_) and H_2_O_2_ (FE_H2O2_) for BVO_cOIHP devices were calculated (Figure 3d; Figures S11–S13, Supporting Information).^[^ ^46^ ^]^ The calculated FE bar chart clearly proved that the BVO_cOIHP promotes ^3^O_2_ production while simultaneously inhibiting the competitive by‐product of H_2_O_2_, thereby accelerating the OER kinetics. The FE_H2O2_ of BVO_*S*‐2F‐MBA (6.65%) was slightly lower than BVO_*S*‐MBA devices (9.82%), indicating the sluggish reaction was more effectively restricted in BVO_*S*‐2F‐MBA devices. The statistical results derived from the 10 devices further corroborated that the average V_onset_ of the BVO_S‐2F‐MBA (≈0.33 V_RHE_) was cathodically shifted ≈50 mV compared to the BVO_*S*‐MBA (≈0.38 V_RHE_), as shown in Figure S16 (Supporting Information). In addition, the average photocurrent density of BVO_*S*‐2F‐MBA devices (4.31 mA cm⁻^2^) showed considerable improvement over that of the BVO_*S*‐MBA devices (3.08 mA cm⁻^2^). Consequently, the BVO_*S*‐2F‐MBA device not only exhibited a higher ABPE of 1.39% at a lower potential compared to 0.85% for BVO_S‐MBA (Figure 2d), but also achieved a maximum IPCE of 89% at 372 nm, notably >75.4% observed from the BVO_*S*‐MBA device (Figure 2e).

Interestingly, we found that the photocurrent density of the BVO_*S*‐2F‐MBA device outperformed the BVO_*S*‐MBA device despite the less favorable band alignment for hole extraction from n^+^‐type BVO to n‐type *S*‐2F‐MBA cOIHP (Figure 2b). This behavior contradicts the expectation based on the energy band diagram, where the p‐type *S*‐MBA cOIHP and n^+^‐type BVO interface should provide optimal conditions for hole extraction to the catalytic active site. This observation suggests that other factor beyond band alignment consideration significantly contributes to the superior OER efficiency on the BVO_*S*‐2F‐MBA device. According to the CISS measurement result that represents the injected charge carriers with different spin states (Figure 3a,b), the *I*_down_ values at −1.8 V showed a significant increase from −39.6 nA in *S*‐MBA cOIHPs to −249 nA in *S*‐2F‐MBA cOIHP. This result confirms that the enhanced photocurrent density in the BVO_*S*‐2F‐MBA photoanode is considerably ascribed to the fact that *S*‐2F‐MBA cOIHP more effectively manipulates spin‐polarized hole transport in the out‐of‐plane direction than *S*‐MBA cOIHP.^[^ ^37^ ^]^

**Figure 4:**
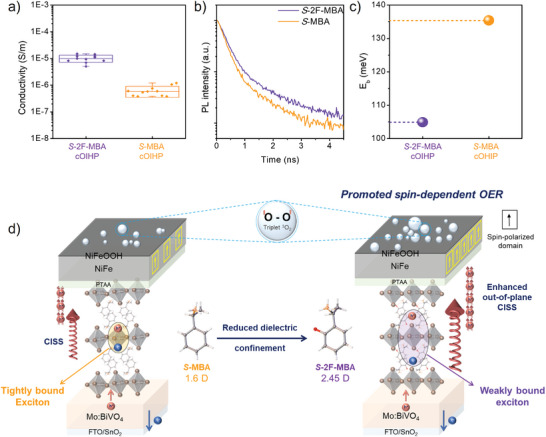
a) Out‐of‐plane conductivity measured from the device structure of FTO/cOIHP(≈730 nm)/Au(70 nm). The plot shows statistical results obtained from the 10 distinct electrodes (area = 0.09 cm^2^). The conductivity was calculated using the following equation: $R=\frac{I}{V}=\rho \frac{L}{A}$, $\sigma \;=\frac{1}{\rho }$, where ρ, L, A, and σ represent resistivity, thickness of the thin film, electrode area, and conductivity, respectively. b) TRPL spectroscopy of the cOIHP thin films on soda‐lime glass measured with an excitation wavelength of 375 nm. c) Plot of exciton binding energy extracted from the cryogenic temperature to room temperature PL measurement. d) Schematic illustration of enhanced out‐of‐plane conductivity and CISS effect in *S*‐2F‐MBA cOIHP, achieved by reducing the dielectric confinement effect, leading to improved spin‐dependent OER.

As shown in Figure S17 (Supporting Information), the *I*_0_ values, which represent out‐of‐plane charge transport without spin polarization measured when the Ni electrode was unmagnetized, also substantially increased from *S*‐MBA cOIHPs (*I*_0_ of −2.98 nA) to *S*‐2F‐MBA cOIHP (*I*_0_ of −19.7 nA) at −1.8 V. Interestingly, the simultaneous increment of both *I*_0_ and *I*_down_ suggested that the carrier transport ability across cOIHP spin polarizer varies depending on the type of the chiral cations. Therefore, we quantitatively analyzed the out‐of‐plane conductivity using FTO/cOIHP/Au device configuration.^[^ ^40^, ^47^ ^]^ As shown in **Figure** **4**a, the *S*‐2F‐MBA cOIHP exhibited ≈15‐fold higher conductivity of 9.79 × 10^−6^ S m⁻^1^ compared to *S*‐MBA cOIHP (6.24 × 10^−7^ S m⁻^1^). To further assess the carrier dynamics within cOIHP, time‐resolved photoluminescence (TRPL) spectroscopy was performed (Figure 4b and **Table** **1**).^[^ ^48^ ^]^ The fast (τ_1_) and slow (τ_2_) decay components were extracted using the bi‐exponential decay function to represent the non‐radiative and radiative recombination lifetime, respectively. Although the τ_1_ showed nearly identical values of ≈0.3 ns for both cOIHPs, the τ_2_ was considerably varied according to the type of chiral cation with longer 2.91 ns for *S‐*2F‐MBA cOIHP compared to 2.08 ns for *S*‐MBA cOIHP. Therefore, the prolonged carrier lifetime inside the *S*‐2F‐MBA cOIHP results in a higher conductivity relative to the *S*‐MBA cOIHP.^[^ ^49^ ^]^

**Table 1:** Result of bi‐exponential fitting of TRPL decay curves and the parameters.

	PL peak λ [nm]	A_1_ [%]	τ_1_ [ns]	A_2_ [%]	τ_2_ [ns]	τ_avg_ [ns]
*S*‐2F‐MBA	530	92.79745	0.33	7.202548	2.91	1.38325
*S*‐MBA	526	93.94696	0.29	6.053024	2.08	0.85575

In principle, in the 2D layered OIHP structure, the varying carrier dynamics are attributable to changes in the exciton binding energy (E_b_). Therefore, the E_b_ of the 2D cOIHP was extracted from temperature‐dependent PL spectra as shown in Figure 4c (see details in Figure S18 and Note S1, Supporting Information).^[^ ^26^ ^]^ Due to the pronounced quantum confinement effect resulting from the reduced dimension compared to 3D counterpart, both cOIHPs displayed huge E_b_ over 100 meV. However, the *S*‐2F‐MBA cOIHP showed a relatively lower E_b_ of 104.9 meV, whereas the *S*‐MBA cOIHP exhibited a considerably larger E_b_ of 135.4 meV. This strongly suggests that the E_b_ sensitively varies depending on the chiral cation composition, resulting in a significant impact on the carrier dynamics. The 2D cOIHP structure is comprised of alternating layers: organic barriers with a smaller dielectric constant (ε_organic_) and inorganic octahedra with a higher dielectric constant (ε_inorganic_).^[^ ^24^ ^]^ Such a significant dielectric constant mismatch induces an extra dielectric confinement effect within the 2D cOIHP lattice. In this context, the E_b_ can be easily modulated by changing the organic component since E_b_ is inversely proportional to the ε_organic_ according to Equation (2):

(2) $$E_{b}\;\propto \;(\frac{\varepsilon_{\;\mathrm{inorganic}}}{\varepsilon_{\;\mathrm{organic}}})$$

Thus, the most electronegative fluorine can increase the polarity of the organic molecules, modifying their surrounding dielectric environment. Therefore, the dipole moments of 2D cOIHP before and after fluorination were predicted using density functional theory (DFT) calculation.^[^ ^30^, ^31^, ^50^ ^]^ The dipole moment of *S‐*2F‐MBA, measured at 2.45 D, is distinctively higher than that of *S*‐MBA (1.60 D), aligning well with the previous report. The DFT calculation confirms that the fluorination of the chiral cation reduces organic–inorganic dielectric constant mismatch, improving Coulomb screening between electrons and holes within the inorganic layers.^[^ ^30^ ^]^ Hence, the *S*‐2F‐MBA cOIHP can further facilitate the out‐of‐plane conductivity inside the cOIHP due to weakly bound exciton.^[^ ^27^, ^47^ ^]^ To confirm the carrier dynamics depending upon the varying E_b_, we further conducted PL analysis using an HTL‐only device with a configuration of quartz/BVO/cOHIP/PTAA (hereafter, BVO_cOHIP_PTAA, Figure S19, Supporting Information). The PL intensity of the BVO_*S*‐2F‐MBA_PTAA was noticeably reduced compared to that of the BVO_*S*‐MBA_PTAA, suggesting that the *S*‐2F‐MBA cOIHP can more efficiently extract photogenerated holes from BVO to the catalytic surface because of less dielectric confinement effect. On the contrary, the *S*‐MBA cOIHP dielectrically confines the charge carriers within the multi‐quantum well structure rather than extracting them to the catalytic surface. As demonstrated by the TRPL results based on the BVO_cOIHP_PTAA architecture (Figure S19d and Table S2, Supporting Information), the BVO_*S*‐2F‐MBA_PTAA exhibited faster PL decay than BVO_*S*‐MBA_PTAA, well‐matching with the steady‐state PL results. Furthermore, it was previously proposed that the exciton spin relaxation rate in 2D OIHP has a quadratic dependence on E_b_ at room temperature.^[^ ^51^ ^]^ Therefore, in the case of *S‐*2F‐MBA cOIHP, the slightly higher *P*_spin_ than that of *S*‐MBA cOIHP (Figure 3a,b) can be ascribed to the diminished E_b_, which leads to a slow spin relaxation rate.^[^ ^51^, ^52^ ^]^ Consequently, the fluorination strategy in the chiral cation promotes the spin‐dependent carrier transport dynamics in 2D cOIHP by lowering the dielectric confinement effect. As a result, the BVO_*S*‐2F‐MBA device outperforms the BVO_*S*‐MBA device due to the predominantly enhanced spin‐polarized hole conductivity within the *S*‐2F‐MBA cOIHP, overcoming the less favorable band alignment (Figure 2b).

Figure 4d comprehensively elucidates how the fluorination of chiral cation in cOIHP can enhance the spin‐dependent OER. The spin state of the photogenerated holes transferred from the BVO can be effectively polarized into a specific direction through the CISS effect in cOIHP spin polarizer. Then, the spin‐polarized holes arriving at the ferromagnetic NiFe layer can generate a specific direction of net magnetization,^[^ ^53^ ^]^ thereby spin pinning occurs at the NiFeOOH catalytic site.^[^ ^49^ ^]^ Specifically, in the BVO_*S*‐2F‐MBA devices, *S*‐2F‐MBA cOIHP induces the spin‐polarization of the active site because of the significantly enhanced conductivity and higher *P*_spin_ of 88.9% compared to 86.6% of S‐MBA cOIHP, originating from the reduced dielectric confinement effect. Therefore, the improved spin alignment as well as increased injected hole density at the NiFeOOH active site effectively boosts the spin‐dependent OER kinetics.^[^ ^54^ ^]^

### 2.4 Enhanced Operational Stability Stemming from the Fluorination Chiral Cation

**Figure 5:**
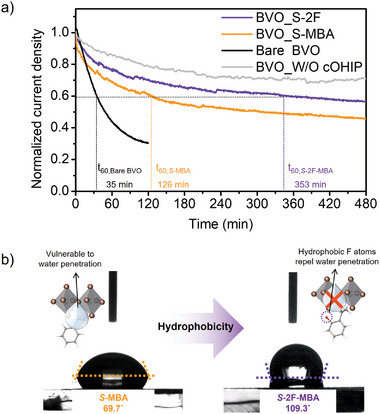
a) Normalized photocurrent density decay spectra and the position of t_60_, which represents the time when the photocurrent degrades to 60% of initial photocurrent density. Bare BVO represents FTO/SnO_2_/BVO (*i.e*, bare BVO) and the BVO_W/O cOHIP device configuration is FTO/SnO_2_/BVO/PTAA/NiFe/NiFeOOH (i.e., BVO_W/O cOHIP). The stability test was conducted under 1.0 m K‐Bi electrolyte (pH 9.0). b) Contact angle result of the *S*‐MBA and *S*‐F‐MBA structural isomer‐based cOIHP thin films on the glass substrate.

Finally, the operational stability of the spin‐dependent OER devices was evaluated, as shown in **Figure** **5**a. Noticeably, the BVO_*S*‐MBA device exhibited t_60_ = 126 min, which represents the time at which the photocurrent density drops to 60% relative to its initial value (refer to Figure S20 for unnormalized data, Supporting Information). This reasonable durability over 2 h in a polar electrolyte without any encapsulation evidently supports the structural integrity of the embedded cOHIP spin polarizer, owing to the introduction of a 16 nm‐thick NiFe protective layer as well as a well‐adhered electrodeposited NiFeOOH catalyst. More interestingly, the BVO_*S‐*2F‐MBA device experienced slower degradation relative to the BVO_*S*‐MBA, demonstrating an exceptionally prolonged operational lifetime with a t_60_ of ≈6 h (353 min). This result suggested that the operational stability is closely dependent on the type of spin polarizer. Since the enhanced OER performance primarily stems from the CISS effect of the cOIHP spin polarizer (Figure 4d), ensuring the resistance to water penetration cOIHP is crucial for guaranteeing sustainability. The photograph in Figure S21 (Supporting Information) showed that the NiFe/NiFeOOH catalytic layer at the front side is almost completely preserved after the stability test. However, when viewed from the backside, degradation of cOIHP layer began from the active site (i.e., color was changed into yellow). The degraded cOIHP may result from the penetration of water through microscopic pinholes in the NiFe physical barrier during a long‐term stability test, highlighting the importance of ensuring the cOIHP stability against polar water molecules. For better comparison, we evaluated the operational stability using a bare BVO photoanode without catalyst, which exhibited poor operational stability of t_60_ of 35 min. This poor durability may be attributed to the significant production of highly reactive H_2_O_2_, as indicated by the high FE_H2O2_ in Figure 3d, subjected to corrosion of the BVO photoanode.^[^ ^17^ ^]^ Therefore, we further conducted the stability test using a device without cOIHP layer, with a configuration of FTO/SnO_2_/BVO/PTAA/NiFe/NiFeOOH (hereafter referred to as BVO_W/O cOHIP). As shown in the LSV curve in Figure S22 (Supporting Information), the fill factor and photocurrent density were slightly improved to 2.35 mA cm^−2^ at 1.23 V_RHE_, compared to the bare BVO (2.01 mA cm^−2^ at 1.23 V_RHE_) because the NiFeOOH catalytic layer can enhance the OER kinetics. Notably, the BVO_W/O cOIHP device showed improved durability by sustaining its initial photocurrent density of 70% after 480 min, indicating that the introduction of PTAA/NiFe/NiFeOOH layers can effectively protect the embedded BVO photoanode (Figure 5a; Figure S20, Supporting Information). These observations strongly suggest that the decline in photocurrent density of the BVO_cOIHP spin‐dependent OER device is primarily attributed to the degradation of cOHIP layers, rather than the corrosion of the BVO photoanode or deterioration of the NiFe/NiFeOOH catalytic layer.

Therefore, to confirm the intrinsic hydrophobicity of the cOHIP layers, we conducted contact angle analysis for the cOHIP thin films using deionized (D.I) water. Figure 5b reveals that the contact angle for *S*‐2F‐MBA cOIHP of 109.3° is extremely higher than the angle of 69.7° for S‐MBA cOIHP. This increased contact angle of *S*‐2F‐MBA cOIHP results from the inherently hydrophobic nature of fluorine, representing that the fluorination of organic molecules can substantially reduce water susceptibility.^[^ ^30^, ^55^ ^]^ Therefore, the increased hydrophobicity of the chiral organic cations via fluorination endows extra water repellency to the *S*‐2F‐MBA cOIHP by impeding water penetration into the organic layers. To characterize the stability of the spin‐dependent OER devices, we traced the LSV curves at 30 min intervals during the potentiostatic measurement to estimate the stability and performance changes following the OER reaction (Figure S23a,b, Supporting Information). Both BVO_*S*‐2F‐MBA and BVO_*S*‐MBA spin‐dependent OER devices exhibited a continuous decrease in photocurrent density, with a slight increase in the onset of approximately 0.1V_RHE_ after a 60 min potentiostatic analysis. However, a noticeable difference between the BVO_*S*‐2F‐MBA and BVO_*S*‐MBA devices emerged after 90 min from in terms of the LSV curve shape. Specifically, the BVO_*S*‐MBA device experienced a considerable loss in fill factor as well as photocurrent density, whereas the BVO_S‐2F‐MBA maintained a consistent LSV shape up to 240 min despite a decrease in photocurrent density. The LSV results traced along the stability test strongly indicated that the *S*‐2F‐MBA cOIHP spin polarizer layer was well‐remained after a long‐term OER reaction. In contrast, the *S*‐MBA cOIHP layer rapidly deteriorated after 90 min due to the hygroscopic nature of the *S*‐MBA cation, leading to significant performance degradation. Digital photographs of the substrates taken from the backside further support the LSV tracking results, showing that the *S*‐2F‐MBA cOIHP spin polarizer remained intact, whereas the *S*‐MBA cOIHP spin polarizer significantly degraded after 4 h of measurement (Figure S23c,d, Supporting Information). Moreover, the stability test of the cOIHP thin films under humid conditions (RH 85%) without any encapsulation further substantiates the water durability of the *S*‐2F‐MBA cOIHP (Figure S24, Supporting Information). The *S*‐2F‐MBA cOIHP remained 44% of its initial (002) peak intensity after 7 days under RH 85%, demonstrating that its 2D cOHIP layered structure was notably durable under a highly humid environment, whereas the 2D structure of the *S*‐MBA cOHIP completely disappeared during the same duration. Consequently, the improved water stability of the *S*‐2F‐MBA cOHIP spin polarizers significantly contributes to the extended operational stability of the spin‐dependent OER device (Figure 5a).

## 3 Conclusion

In summary, we report that novel fluorinated chiral cation can dramatically enhance the spin‐polarized charge carrier conductivity and water‐stability of OHIP spin polarizer. Especially, the fluorination approach significantly increases the dielectric constant of the chiral cations, thereby mitigating the dielectric constant disparity between organic and inorganic layers. Owing to the alleviated MQW structure, the excitons are effectively capable of dissociation into free carriers while maintaining their aligned spin‐state within the cOIHPs, resulting in facilitated spin‐polarized carrier transport. By virtue of this novel spin polarizer, the BVO photoanode embedded with *S‐*2F‐MBA cOIHP spin polarizer achieves a high photocurrent density through efficient spin‐dependent OER at the catalytic surface. Additionally, the photoanode featuring the novel S‐2F‐MBA cOIHP exhibits better long‐term operational stability, with a t_60_ of ≈6 h in polar electrolytes. This stability is attained even in the absence of superhydrophobic encapsulation, thanks to the reinforced resistance to water intrusion by the spin polarizer. Our strategy uniquely boosts the OER performance by effectively polarizing the spin state of the charge carriers through fluorinated cOIHP. This proposed material design principle will open new possibilities for developing an ideal cOIHP spin polarizer for other spin‐dependent multi‐electron chemical reactions.

## 4 Experimental Section

### Materials

(*S/rac*)−1‐(2‐fluoromethyl)benzylamine (> 98%) was purchased from Aaron Chemicals. Lead iodide (99.99%) was purchased from TCI Chemicals. (*S,rac*)‐α‐methyl benzylamine (> 98%), Lead oxide (99%), hydroiodic acid (57 wt.% in H_2_O, > 99.99%), N, N‐dimethylformamide (DMF, anhydrous, 99.8%), dimethyl sulfoxide (DMSO, anhydrous, 99.9%), Bi(NO_3_)_3_·5H2O (98%), VO(acac)_2_ (98%), MoO_2_(acac)_2_ (98%), and polyethylene glycol (PEG) 200 (molecular mass 190–210) were purchased from Sigma‐Aldrich. All materials were used as received without further purification.

### Synthesis of S‐MBAI and S‐2F‐MBAI Chiral Organic Salts

First, either 1.3 mL of S‐MBA or *S*‐2F‐MBA molecule was dissolved in 3 mL ethanol followed by vigorous stirring for 5 min in the N_2_ glove box. Then, 900 µL of HI was added and the solutions were stirred for 12 h at room temperature. The stirred solution was then fully evaporated at 90 °C in a vacuum oven for 3 days, and the white solid precipitates were washed with diethyl ether several times and dried in a vacuum at 90 °C for 2 days.

### Synthesis of Single Crystals and Single Crystal X‐ray Diffraction

A stoichiometric amount of *S‐*2F‐MBA (1 mmol, 135 µL) and PbO (0.5 mmol, 111.6 mg) were dissolved in a mixture of HI (2.5 mL) and H_3_PO_2_ (0.5 mL) solution. Upon mixing, orange precipitates initially formed and were redissolved to the pale‐yellow solutions under stirring at 120 °C for 30 min. This hot solution was placed in a temperature‐controlled oven and cooled to room temperature at a rate of 1 °C min^−1^, resulting in orange block‐shaped crystals for *S‐*2F‐MBA based cOIHP. The obtained crystals were analyzed using the Bruker SMART APEX‐II (Mo Kα radiation, λ = 0.71073Å). The structural parameters were obtained by SHELXT and refined by SHELXL. The crystallographic structure was visualized using VESTA software.

### Chiral Perovskite Thin Film Fabrication

Stoichiometric amounts of either *S*‐2F‐MBAI salt (80.51 mg) or *S*‐MBAI salt (77.91 mg) and PbI_2_ (69.49 mg and 72.01 mg for S‐2F‐MBA and *S*‐MBA cOIHP, respectively) were dissolved in a mixture of DMF (628 µL) and DMSO (15 µL) in N_2_ glovebox to make 20 wt.% concentration. After stirring for 6 h at room temperature, the precursor was filtrated by a 0.2‐µm syringe filter, followed by spin‐coating at 2000 rpm for 30 s onto a UV‐ozone‐treated 2 cm ×  2 cm FTO substrate that was cleaned via sequential sonication with deionized water, acetone, and ethanol in a sonicator (15 min for each process). The spin‐coated films were annealed for 15 min at 80 °C. The spin‐coating and annealing procedures were conducted in a dry room (relative humidity (RH) < 20%).

### Fabrication of cOIHP Spin Polarizer Embedded Photoanode and PEC Characterizations

For the synthesis of BVO photoabsorber, 56.4 mg SnCl_2_·2H_2_O was dissolved in 5 mL isopropyl alcohol to prepare SnO_2_ precursor. The precursor solution was spin‐coated on the UV‐ozone‐treated 3 cm  ×  3 cm FTO substrates at 2000 rpm for 30 s followed by annealing at 500 °C for 30 min. For the Mo‐doped BVO, 0.075 mol of MoO_2_(acac)_2_ was added to 5 mL methanol, and 1212.7 mg Bi(NO_3_)_3_·5H_2_O was dissolved in 5 mL glacial acetic acid (Duksan Pure Chemicals, Korea). The 55 µL Mo‐doping solution and 0.75 mL Bi(NO_3_)_3_·5H_2_O solution were mixed with the solution containing 99.4 mg VO(acac)_2_ in 5 mL methanol for 1at% Mo‐doped BVO. The prepared solution was filtered by a 0.2‐µm syringe filter, and 180 µL of the solution was spin‐coated onto the prepared FTO/SnO_2_ substrate at 2000 rpm 20 s. After spin coating, the substrates were immediately placed on a hot plate at 300 °C for 2 min and then further annealed at 400 °C for 3 min. The deposition/annealing cycles were repeated five times for a 150‐nm‐thick Mo‐doped BVO photoabsorber, followed by final annealing at 480 °C for 2 h in a box furnace. For photoanode fabrication with the BVO/cOIHP/PTAA/NiFe/NiFeOOH device structure, the 20 wt.% cOIHP precursor was spin‐coated on the prepared BVO absorber at 2000 rpm for 30 s and annealed for 15 min at 65 °C. Poly[bis(4‐phenyl)(2,4,6‐trimethylphenyl)amine] (PTAA, Ossila) precursor dissolved in chlorobenzene of 6 mg mL^−1^ was spin‐coated on BVO/cOIHP at 5000 rpm for 30 s and annealed at 60 °C for 10 min. Then, the 16‐nm‐thick NiFe (Ni: Fe = 78wt.%:22wt.%) was deposited by thermal evaporation. After the Cu wire was contacted to the FTO/SnO_2_ electrode by silver paste, the surface of the device was covered by epoxy resin excluding an active area of 0.09 cm^2^. For the deposition of NiFeOOH co‐catalyst, 40 mg NiSO_4_·6H_2_O and 120 mg FeSO_4_·7 H_2_O were dissolved in 400 mL of 0.5 m potassium hydrogen carbonated aqueous solution. Before use, the prepared catalyst precursor was purged with Ar gas for 30 min. The electrodeposition was conducted 20 times with a bias from −0.3 to 0.5 V against the reference electrode (Ag/AgCl/KCl) accompanying pre‐treatment of −0.3 V for 5 s at each cycle. After deposition, the photoanode was washed with copious amounts of deionized water and dried at 50 °C for 1 h. The PEC measurements were conducted under back‐illumination using a potentiostat (SI 1287, Solartron, UK) in a three‐electrode system with Ag/AgCl/KCl (4 m) and Pt coil as the reference and counter electrode, respectively. To compare the PEC performance, the potentials applied on the photoanode were converted to an RHE scale based on the following Equation (3):

(3) $$E_{\mathrm{RHE}}=\;E_{\mathrm{Ag}/\mathrm{AgCl}}\;+\;0.0591\;\mathrm{pH}\;+\;0.197$$

### Fabrication of Device for CISS Phenomenon Confirmation

First, a 50‐nm‐thick nickel layer was deposited by thermal evaporation on the pre‐cleaned soda‐lime glass. After UV‐ozone treatment for 15 min, 1.5‐nm‐thick Al_2_O_3_ spin tunneling buffer layer was deposited on the Ni substrate using atomic layer deposition (ALD) by performing 35 cycles at 110 °C, and the cOIHP thin‐film was deposited at 2000 rpm for 30 s and annealed at 80 °C for 15 min. Then, 8 mg bathocuproine (BCP, TCI Chemicals) was dissolved in 1 mL chlorobenzene at 35 °C, and the resulting BCP precursor was deposited on the cOIHP thin film at 5000 rpm for 30 s. Next, MoO_3_ (15 nm)/Al (100 nm) layer was deposited by thermal evaporation. The I‐V curve was measured after pre‐magnetization by a strong permanent magnet (> 5000 Gauss).

### Fabrication of Device for Out‐of‐Plane Conductivity Measurement

For out‐of‐plane conductivity measurement device with a configuration of the FTO/cOIHP/Au, stoichiometric amounts of either *S*‐2F‐MBAI salt (483.06 mg) or *S‐*MBAI salt (467.46 mg) and PbI_2_ (416.94 and 432.06 mg for *S*‐2F‐MBA and *S*‐MBA cOHIP, respectively) were dissolved in a mixture of DMF (942 µL) and DMSO (22.5 µL) to make 50 wt.% concentration in N_2_ glovebox. After thin‐film fabrication, the 70 nm Au layer was deposited by a thermal evaporator through a shadow mask with an area of 0.09 cm^2^. The out‐of‐plane conductivity was measured using a semiconductor parameter analyzer (Agilent 4155C) in a probe station.

### Characterizations

The surface and side‐view morphologies were investigated by field emission scanning electron microscopy (FE‐SEM, JSM‐7001F, JEOL Ltd). The CD data were recorded using a CD spectrometer (J‐815, JASCO). The PL and TRPL spectra were collected with an excitation beam wavelength of 375 nm (FluoroMax, Horiba, Japan). The XRD patterns were obtained with an XRD instrument (Rigaku Miniflex 600, The Woodlands) using Cu Kα radiation. The XRD patterns were calibrated with FTO peaks at 26.68°. The contact angle measurements were performed using a Phoenix‐Alpha contact‐angle system (SEO, Korea). The UPS (AXIS‐NOVA, and Ultra DLD, UK) for the energy level measurement was analyzed under He I radiation at 21.21 eV. The IPCE analyses were conducted using an electrochemical workstation (Zennium, Zahner). The ABPE was calculated using the LSV curve according to Equation (4):

(4) $$\mathrm{ABPE}\;\left(\%\right)\;=\;\frac{\mathrm{Photocurrent}\;\mathrm{density}\;\times \left(1.23-V_{\mathrm{RHE}}\right)}{P_{0}}\times \;100\%$$

where *P*_0_ represents the obtained total light intensity of AM 1.5G (100 mW cm⁻^2^). To detect H_2_O_2_, 1 mL *o*‐tolidine (Duksan Pure Chemicals, Korea) and 3 mL of 0.1 m Na_2_SO_4_ electrolyte retrieved after 1 h reaction was mixed. If the H_2_O_2_ is present, the solution turns yellow. The absorption spectra were obtained immediately after mixing using UV‐vis absorption spectroscopy (V‐670, JASCO). The theoretical amount of O_2_ gas was calculated from Faraday's law (equation 5) and O_2_ evolution was evaluated using an optical fluorescence sensor (NeoFox, Ocean Optics). The cell was purged with Ar before the measurement:

(5) $$n\;=\;\frac{I\;\times \;t}{z\;\times \;F}$$

where *n* is the number of O_2_ molecules, *I* is the current in ampere, *t* is the time in seconds, *z* is the number of electron transfers during formation (i.e., O_2_ for 4 and H_2_O_2_ for 2), and F is the Faraday constant (96 485 C mol^−1^).

### DFT Calculation

We employed an exchange‐correlation functional parametrized by Perdew, Burke, and Ernzerhof.^[^ ^56^ ^]^ The plane waves were expanded up to 400 eV for self‐consistent field calculations. After the cell optimization, the plane waves were expanded up to 400 eV for self‐consistent field calculations.

## Conflict of Interest

The authors declare no conflict of interest.

## Supporting information

Supporting Information

## Data Availability

The data that support the findings of this study are available from the corresponding author upon reasonable request.

## References

[1] A. M. K. Fehr , A. Agrawal , F. Mandani , C. L. Conrad , Q. Jiang , S. Y. Park , O. Alley , B. R. Li , S. Sidhik , I. Metcalf , C. Botello , J. L. Young , J. Even , J. C. Blancon , T. G. Deutsch , K. Zhu , S. Albrecht , F. M. Toma , M. C. Wong , A. D. Mohite , Nat. Commun. 2023, 14, 3797. 37365175 10.1038/s41467-023-39290-y PMC10293190

[2] L. Li , J. Zhou , X. Wang , J. Gracia , M. Valvidares , J. Ke , M. M. Fang , C. Q. Shen , J. M. Chen , Y. C. Chang , C. W. Pao , S. Y. Hsu , J. F. Lee , A. Ruotolo , Y. Chin , Z. W. Hu , X. Q. Huang , Q. Shao , Adv. Mater. 2023, 35, 2302966. 10.1002/adma.202302966 37436805

[3] K. Sivula , R. Krol , Nat. Rev. Mater. 2016, 1, 16010.

[4] X. Ren , T. Z. Wu , Y. M. Sun , Y. Li , G. Y. Xian , X. H. Liu , C. M. Shen , J. Gracia , H. J. Gao , H. T. Yang , Nat. Commun. 2021, 12, 2608. 33972558 10.1038/s41467-021-22865-y PMC8110536

[5] Y. C. Liang , M. Lihter , M. Lingenfelder , Isr. J. Chem. 2022, 62, 20220005.

[6] S. Ghosh , B. P. Bloom , Y. Y. Lu , D. Lamont , D. H. Waldeck , J. Phys. Chem. 2020, 124, 22610.

[7] W. Mtangi , V. Kiran , C. Fontanesi , R. Naaman , J. Phys. Chem. Lett. 2015, 6, 4916. 26615833 10.1021/acs.jpclett.5b02419 PMC4685426

[8] F. A. Garcés‐Pineda , M. Blasco‐Ahicart , D. Nieto‐Castro , N. López , J. R. Galán‐Mascarós , Nat. Energy 2019, 4, 519.

[9] W. Q. Gao , R. Peng , Y. Y. Yang , X. L. Zhao , C. Cui , X. W. Su , W. Qin , Y. Dai , Y. D. Ma , H. Liu , ACS Energy Lett. 2021, 6, 2129.

[10] A. Vadakkayil , C. Clever , K. N. Kunzler , S. S. Tan , B. P. Bloom , D. H. Waldeck , Nat. Commun. 2023, 14, 1067. 36828840 10.1038/s41467-023-36703-w PMC9958132

[11] W. Y. Zhang , K. Banerjee‐Ghosh , F. Tassinari , R. Naaman , ACS Energy Lett. 2018, 3, 2308.

[12] K. B. Ghosh , W. Y. Zhang , F. Tassinari , Y. Mastai , O. Lidor‐Shaley , R. Naaman , P. Möllers , D. Nürenberg , H. Zacharias , J. Wei , E. Wierzbinski , D. H. Waldeck , J Phys Chem 2019, 123, 3024.

[13] H. P. Lu , Z. V. Vardeny , M. C. Beard , Nat. Rev. Chem. 2022, 6, 470. 37117313 10.1038/s41570-022-00399-1

[14] W. Mtangi , F. Tassinari , K. Vankayala , A. V. Jentzsch , B. Adelizzi , A. R. A. Palmans , C. Fontanesi , E. W. Meijer , R. Naaman , J. Am. Chem. Soc. 2017, 139, 2794. 28132505 10.1021/jacs.6b12971 PMC5330654

[15] R. Naaman , Y. Paltiel , D. H. Waldeck , Nat. Rev. Chem. 2019, 3, 250.

[16] K. Michaeli , N. Kantor‐Uriel , R. Naaman , D. H. Waldeck , Chem. Soc. Rev. 2016, 45, 6478. 27734046 10.1039/c6cs00369a

[17] H. Im , S. Ma , H. Lee , J. Park , Y. S. Park , J. Yun , J. Lee , S. Moon , J. Moon , Energy Environ. Sci. 2023, 16, 1797.

[18] M. H. Ai , L. Pan , C. X. Shi , Z. F. Huang , X. W. Zhang , W. B. Mi , J. J. Zou , Nat. Commun. 2023, 14, 4562. 37507418 10.1038/s41467-023-40367-x PMC10382512

[19] J. Y. Wang , B. R. Mao , Z. V. Vardeny , J. Chem. Phys. 2023, 159, 091002. 37675847 10.1063/5.0160032

[20] Y. Lu , Q. Wang , R. Y. Chen , L. L. Qiao , F. X. Zhou , X. Yang , D. Wang , H. Cao , W. L. He , F. Pan , Z. Yang , C. Song , Adv. Funct. Mater. 2021, 31, 2104605.

[21] H. P. Lu , J. Y. Wang , C. X. Xiao , X. Pan , X. H. Chen , R. Brunecky , J. J. Berry , K. Zhu , M. C. Beard , Z. V. Vardeny , Sci. Adv. 2019, 5, eaay0571. 31840072 10.1126/sciadv.aay0571 PMC6897542

[22] H. H. P. Lu , C. X. Xiao , R. Y. Song , T. Y. Li , A. E. Maughan , A. Levin , R. Brunecky , J. J. Berry , D. B. Mitzi , V. Blum , M. C. Beard , J. Am. Chem. Soc. 2020, 142, 13030. 32602710 10.1021/jacs.0c03899

[23] H. Lee , S. Ma , S. Oh , J. W. Tan , C. U. Lee , J. Son , Y. S. Park , J. Yun , G. Jang , J. Moon , Small 2023, 19, 2304166. 10.1002/smll.202304166 37282813

[24] B. Cheng , R. R. Yu , G. S. Xing , Y. L. Wang , W. L. Wang , Y. Chen , X. W. Xu , Q. Zhao , ACS Energy Lett. 2023, 9, 226.

[25] R. Chakraborty , A. Nag , Phys. Chem. Chem. Phys. 2021, 23, 82. 33325476 10.1039/d0cp04682e

[26] R. Chakraborty , A. Nag , J. Phys. Chem. C 2020, 124, 16177.

[27] B. Cheng , T. Y. Li , P. Maity , P. C. Wei , D. Nordlund , K. T. Ho , D. H. Lien , C. H. Lin , R. Z. Liang , X. H. Miao , I. A. Ajia , J. Yin , D. Sokaras , A. Javey , I. S. Roqan , O. F. Mohammed , J. H. He , Commun. Phys. 2018, 1, 80.

[28] J. J. Yoo , S. S. Shin , J. Seo , ACS Energy Lett. 2022, 7, 2084.

[29] X. T. Li , J. M. Hoffman , M. G. Kanatzidis , Chem. Rev. 2021, 121, 2230. 33476131 10.1021/acs.chemrev.0c01006

[30] J. S. Shi , Y. R. Gao , X. Gao , Y. Zhang , J. J. Zhang , X. Jing , M. Shao , Adv. Mater. 2019, 31, 1901673. 10.1002/adma.201901673 31379023

[31] S. Q. Tan , N. Zhou , Y. H. Chen , L. Li , G. L. Liu , P. F. Liu , C. Zhu , J. Z. Lu , W. T. Sun , Q. Chen , H. P. Zhou , Adv. Energy Mater. 2019, 9, 1803024.

[32] M. K. Jana , R. Y. Song , H. L. Liu , D. R. Khanal , S. M. Janke , R. D. Zhao , C. Liu , Z. V. Vardeny , V. Blum , D. B. Mitzi , Nat. Commun. 2020, 11, 4699. 32943625 10.1038/s41467-020-18485-7 PMC7499302

[33] M. K. Jana , R. Y. Song , Y. Xie , R. D. Zhao , P. C. Sercel , V. Blum , D. B. Mitzi , Nat. Commun. 2021, 12, 4682. 34404766 10.1038/s41467-021-25149-7 PMC8371112

[34] J. Ahn , E. Lee , J. Tan , W. Yang , B. Kim , J. Moon , Mater. Horiz. 2017, 4, 851.

[35] Z. X. Zhang , Z. Y. Wang , H. H. Y. Sung , I. D. Williams , Z. G. Yu , H. P. Lu , J Am Chem Soc 2022, 144, 22242. 36399117 10.1021/jacs.2c10309

[36] G. Albano , F. Salerno , L. Portus , W. Porzio , L. A. Aronica , L. Di Bari , ChemNanoMat 2018, 4, 1059.

[37] A. Salij , R. H. Goldsmith , R. Tempelaar , J. Am. Chem. Soc. 2021, 143, 21519. 34914380 10.1021/jacs.1c06752

[38] F. M. Rombach , S. A. Haque , T. J. Macdonald , Energy Environ. Sci. 2021, 14, 5161.

[39] H. M. Zhu , Q. Q. Wang , K. Sun , W. Chen , J. Tang , J. J. Hao , Z. J. Wang , J. Y. Sun , W. C. H. Choy , P. Muller‐Buschbaum , X. W. Sun , K. Wang , ACS Appl Mater Interfaces 2023, 15, 9978. 10.1021/acsami.2c20716 36753711

[40] J. V. Passarelli , D. J. Fairfield , N. A. Sather , M. P. Hendricks , H. Sai , C. L. Stern , S. I. Stupp , J. Am. Chem. Soc. 2018, 140, 7313. 29869499 10.1021/jacs.8b03659

[41] Y. H. Kim , Y. X. Zhai , H. P. Lu , X. Pan , C. X. Xiao , E. A. Gaulding , S. P. Harvey , J. J. Berry , Z. V. Vardeny , J. M. Luther , M. C. Beard , Science 2021, 371, 1129. 33707260 10.1126/science.abf5291

[42] Y. H. Kim , R. Y. Song , J. Hao , Y. X. Zhai , L. Yan , T. Moot , A. F. Palmstrom , R. Brunecky , W. You , J. J. Berry , J. L. Blackburn , M. C. Beard , V. Blum , J. M. Luther , Adv. Funct. Mater. 2022, 32, 2200454.

[43] H. Yang , Y. W. Liu , Y. X. Ding , F. S. Li , L. Q. Wang , B. Cai , F. G. Zhang , T. Q. Liu , G. Boschloo , E. M. J. Johansson , L. Sun , Nat. Commun. 2023, 14, 5486. 37679329 10.1038/s41467-023-41187-9 PMC10484934

[44] G. Righi , J. Plescher , F. P. Schmidt , R. K. Campen , S. Fabris , A. Knop‐Gericke , R. Schlögl , T. E. Jones , D. Teschner , S. Piccinin , Nat. Catal. 2022, 5, 888.

[45] Z. Y. Bian , K. Kato , T. Ogoshi , Z. Cui , B. S. Sa , Y. Tsutsui , S. Seki , M. Suda , Adv. Sci. 2022, 9, 2201063. 10.1002/advs.202201063 PMC9189682 35481673

[46] S. Sultan , M. Ha , D. Y. Kim , J. N. Tiwari , C. W. Myung , A. Meena , T. J. Shin , K. H. Chae , K. S. Kim , Nat. Commun. 2019, 10, 5195. 31729366 10.1038/s41467-019-13050-3 PMC6858335

[47] Y. Zhang , M. Z. Sun , N. Zhou , B. L. Huang , H. P. Zhou , J. Phys. Chem. Lett. 2020, 11, 7610. 32838529 10.1021/acs.jpclett.0c02274

[48] Z. G. Xiao , R. A. Kerner , N. Tran , L. F. Zhao , G. D. Scholes , B. P. Rand , Adv. Funct. Mat. 2019, 29, 1807284.

[49] K. C. Zhang , A. Späth , O. Almora , V. M. Le Corre , J. Wortmann , J. Y. Zhang , Z. Q. Xie , A. Barabash , M. S. Hammer , T. Heumüller , J. Min , R. Fink , N. Li , C. J. Brabec , ACS Energy Lett. 2022, 7, 3235.

[50] Q. H. Li , Y. X. Dong , G. W. Lv , T. T. Liu , D. Lu , N. Zheng , X. Y. Dong , Z. Y. Xu , Z. Q. Xie , Y. S. Liu , ACS Energy Lett. 2021, 6, 2072.

[51] X. H. Chen , H. P. Lu , K. Wang , Y. X. Zhai , V. Lunin , P. C. Sercel , M. C. Beard , J. Am. Chem. Soc. 2021, 143, 19438. 34767709 10.1021/jacs.1c08514

[52] W. J. Tao , Q. H. Zhou , H. M. Zhu , Sci. Adv. 2020, 6, eabb7132.33219022

[53] Z. J. Huang , B. P. Bloom , X. J. Ni , Z. N. Georgieva , M. Marciesky , E. Vetter , F. Liu , D. H. Waldeck , D. L. Sun , ACS Nano 2020, 14, 10370. 32678570 10.1021/acsnano.0c04017

[54] X. A. Ren , T. Z. Wu , Z. Z. Gong , L. L. Pan , J. L. Meng , H. T. Yang , F. B. Dagbjartsdottir , A. Fisher , H. J. Gao , Z. C. J. Xu , Nat. Commun. 2023, 14, 2482. 37120590 10.1038/s41467-023-38212-2 PMC10148796

[55] J. C. Biffinger , H. W. Kim , S. G. DiMagno , ChemBioChem 2004, 5, 622. 15122633 10.1002/cbic.200300910

[56] J. P. Perdew , K. Burke , M. Ernzerhof , Phys. Rev. Lett. 1996, 77, 3865. 10062328 10.1103/PhysRevLett.77.3865

