# Supplementary material for: Fluorinated Organic Cations Derived Chiral 2D Perovskite Enabling Enhanced Spin‐Dependent Oxygen Evolution Reaction
Source: Adv Sci (Weinh). 2024 Jun 28;11(33):2403326. doi: 10.1002/advs.202403326 (PMC11434140; doi:10.1002/advs.202403326)
Supplement: Supplementary file 1 — Supporting Information [file ADVS-11-2403326-s001.docx]

Supporting information for

**Fluorinated Organic Cations Derived Chiral 2D Perovskite Enabling Enhanced Spin-Dependent Oxygen Evolution Reaction**

Jaehyun Son^1^, Gyumin Jang^1^, Sunihl Ma^2^, Hyungsoo Lee^1^, Chan Uk Lee^1^, Seongyeon Yang^1^, Junwoo Lee^1^, Subin Moon^1^, Wooyong Jeong^1^, Jeong Hyun Park^1^, Chan-Woo Jung^3^, Ji-Hee Kim^4^, Ji-Sang Park^5^, and Jooho Moon^1^*

*Corresponding author: jmoon@yonsei.ac.kr

**Table S1.** Crystallographic data and structure refinement for (*S*-2F-MBA)_2_PbI_4_.

| **Empirical formula** | C_16_H_22_F_2_I_4_N_2_ Pb |
| --- | --- |
| **Formula weight** | 995.14 g/mol |
| **Temperature** | 298 K |
| **Wavelength** | 0.71073 Å |
| **Crystal system** | Monoclinic |
| **Space group** | P_21_/n |
| **Unit cell dimensions** | a= 9.30973(16) Å α= 90°  b= 8.92420(14) Å β= 91.5745(14)°  c= 28.8598(4) Å γ= 90° |
| **Volume** | 2396.83(6) Å^3^ |
| **Z** | 4 |
| **Density (calculated)** | 2.758 g/cm^3^ |
| **Absorption coefficient** | 12.209 mm^-1^ |
| **F(000)** | 1776.0 |
| **Crystal size** | 0.200 x 0.100 x 0.040 mm³ |
| **CCDC number** | 2313626 |

**
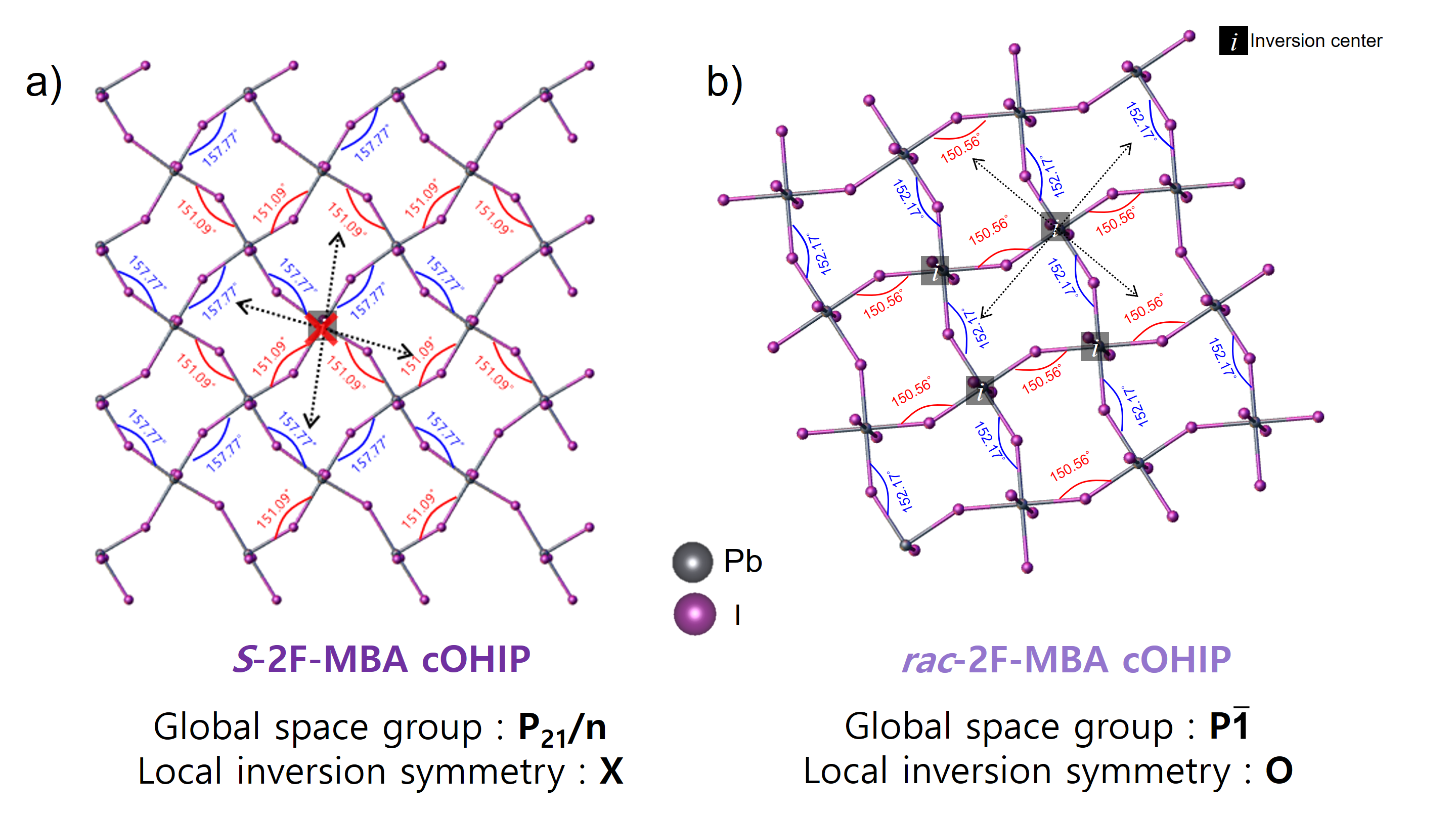
**

**Figure S1.** Inorganic frameworks and distortion angles for a) *S*-2F-MBA cOIHP and b) *rac*-MBA cOHIP. The *rac*-2F-MBA cOIHP was redrawn based on the CCDC number 1893383.^[S1]^ The inorganic frameworks are viewed from the [001] direction, and the C, H, F, and N atoms are omitted for clarity. The black square indicates the inversion symmetry center. Note that the inversion symmetry within the inorganic frameworks in *S*-2F-MBA cOIHP is broken while it is preserved in the *rac*-2F-MBA cOIHP. The crystallographic study demonstrates that the chirality of the *S*-2F-MBA cation is structurally transferred to the local inorganic framework.

**
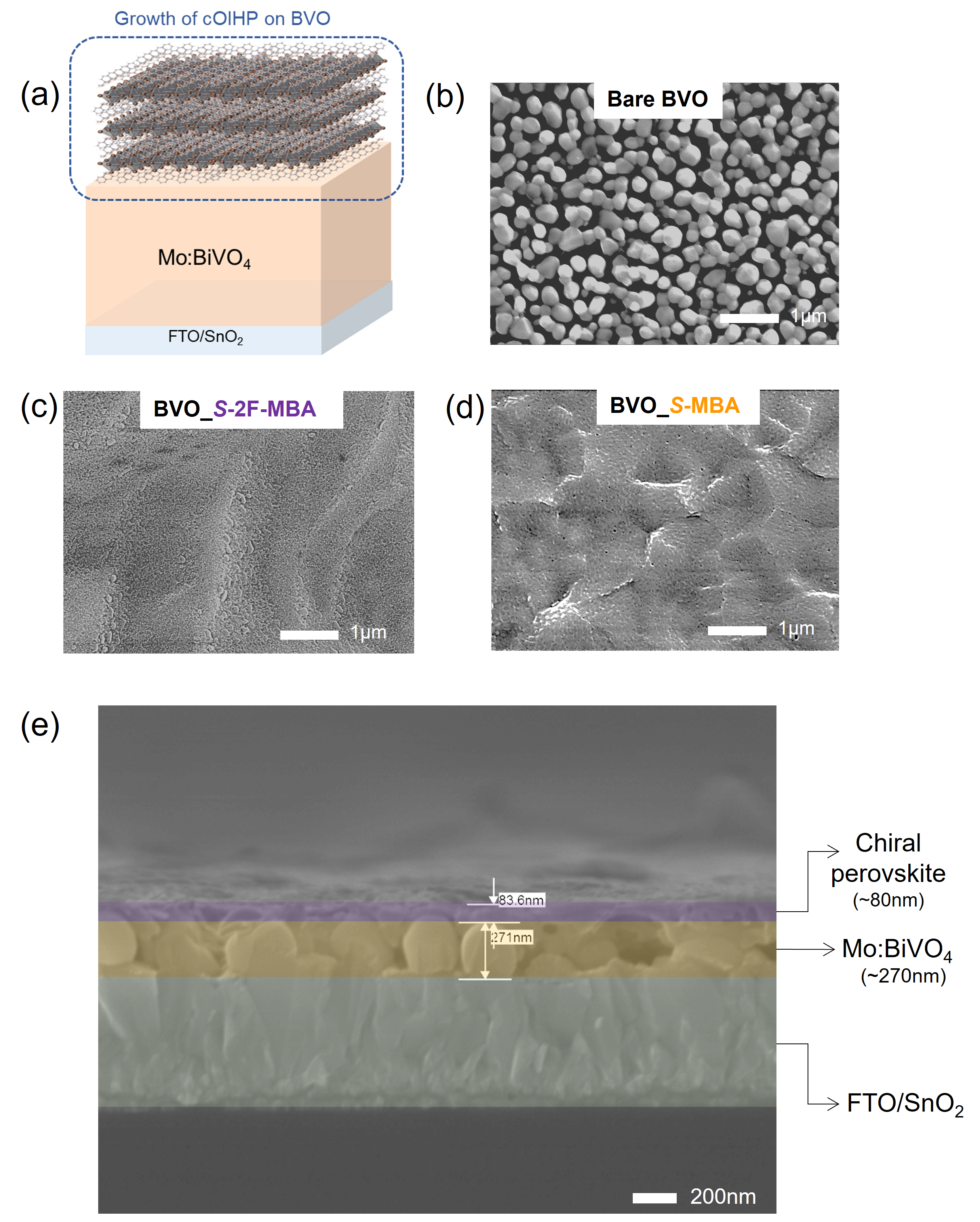
**

**Figure S2.** a) Schematic illustration of the growth of cOIHP on BVO surface. The top surface SEM images of b) bare BVO, c) BVO_*S*-2F-MBA, and d) BVO_*S*-MBA. e) Cross-sectional image of the FTO/SnO_2_/BVO/cOIHP structure. The thickness of cOIHP thin film is approximately 80 nm.

**
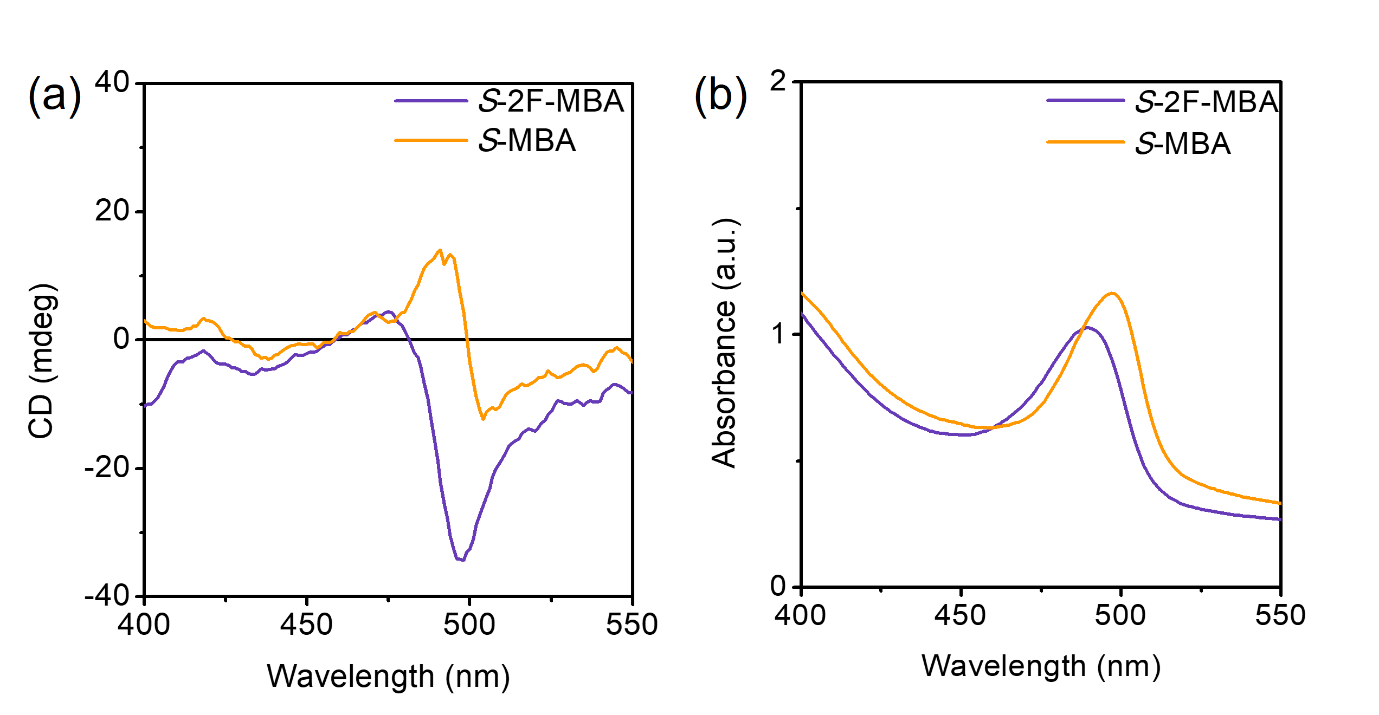
**

**Figure S3.** a) CD spectra of the cOIHP on FTO substrate. b) Absorbance spectra of the cOIHP.

**
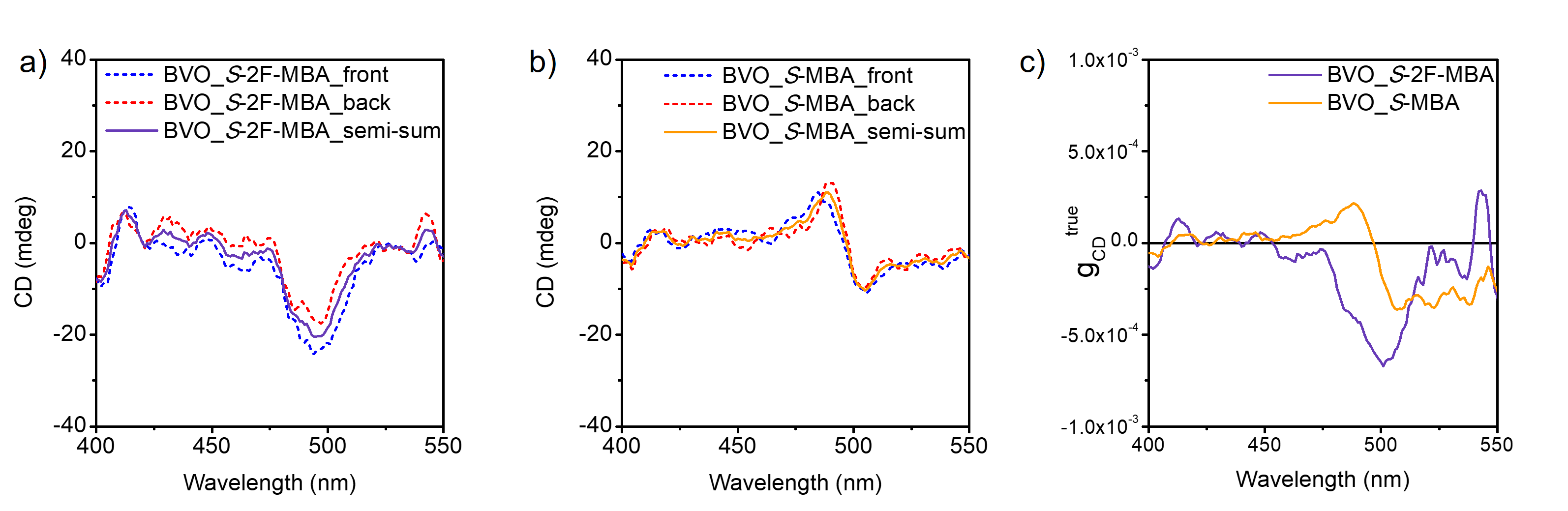
**

**Figure S4.** The bold line was CD_true_ by averaging the CD_front_ (blue dot line) and CD_back_ (red dot line) for a) BVO_*S*-2F-MBA and b) BVO_*S*-MBA, respectively. The anisotropy factor was calculated using the following equation: g_CD_^true^ = $\frac{\text{CD}_{\text{true}}}{\text{32980× Absorbance}}$.

**
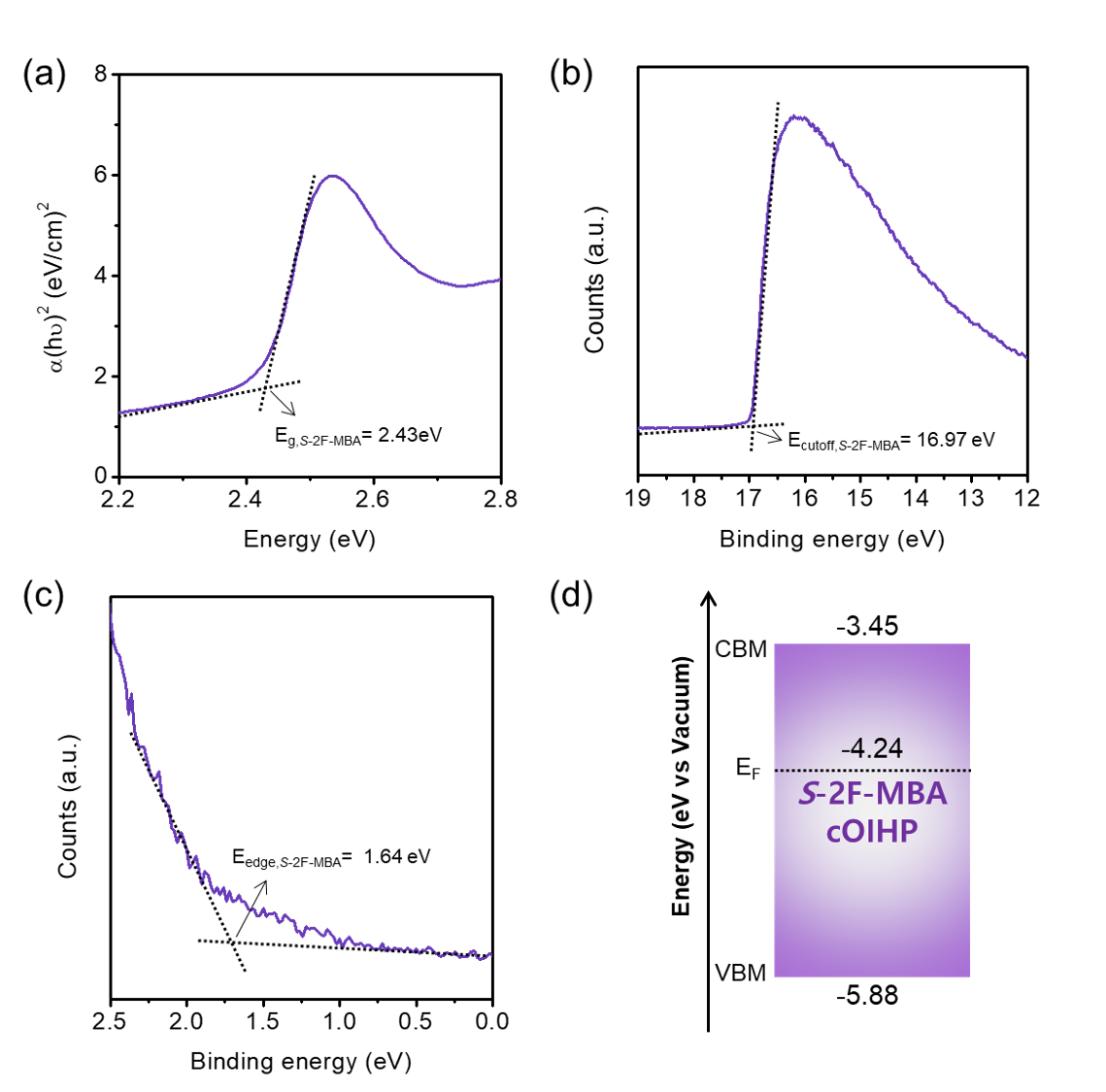
**

**Figure S5.** a) Tauc plot obtained from absorbance spectra of the *S-*2F-MBA cOIHP for calculating bandgap (E_g_). UPS spectra of the b) secondary electron cut-off (E_cut-off_) and c) valence band edge (E_edge_) of the *S*-2F-MBA cOIHP analyzed under He I radiation at 21.21 eV. The Fermi level (E_F_) was calculated by using the following equation: E_F_ = E_cut-off_ – 21.21 eV. Then, the valence band energy (E_VBM_) was calculated by E_VBM_ = E_F_ – E_edge_. d) The calculated energy band diagram of the *S*-2F-MBA cOHIP.

**
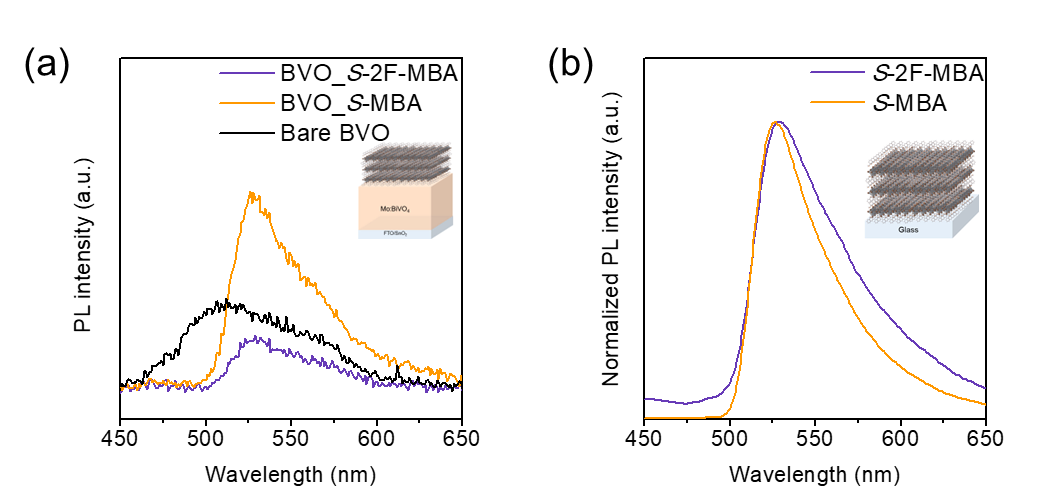
**

**Figure S6.** a) PL spectra obtained from thin films with the configuration of FTO/SnO2/BVO/cOIHP. b) Normalized PL spectra obtained from cOIHP thin films on a soda-lime glass substrate (*i.e.*, glass/cOIHP structure). Note that the PL peak of the BVO_cOIHP is distinctively red-shifted compared to the bare BVO, indicating that the photoexcited holes are transferred from BVO to cOIHP.

**
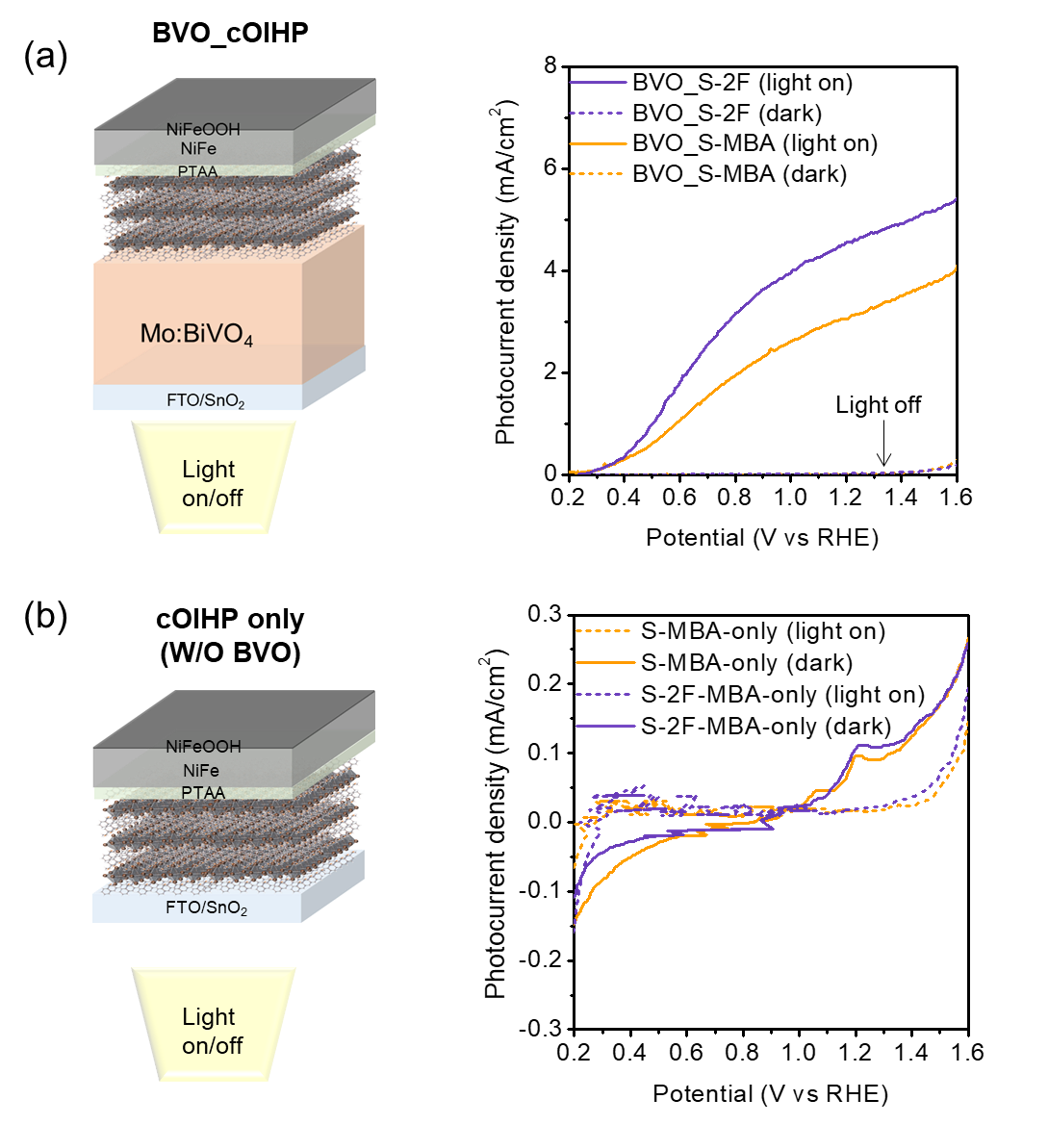
**

**Figure S7.** a) LSV curve for BVO_*S*-2F-MBA and BVO_*S*-MBA devices under back illumination (bold line) and dark condition (dot line), respectively. b) LSV curves for *S*-2F-MBA and *S*-MBA only devices (without BVO) under back illumination (bold line) and dark condition (dot line), respectively. Note that the cOIHP-only devices produce only a minimal photocurrent density of about 0.1 mA/cm^2^ at 1.23 V_RHE_, suggesting negligible photoelectric conversion contribution to the observed photocurrent density.

**
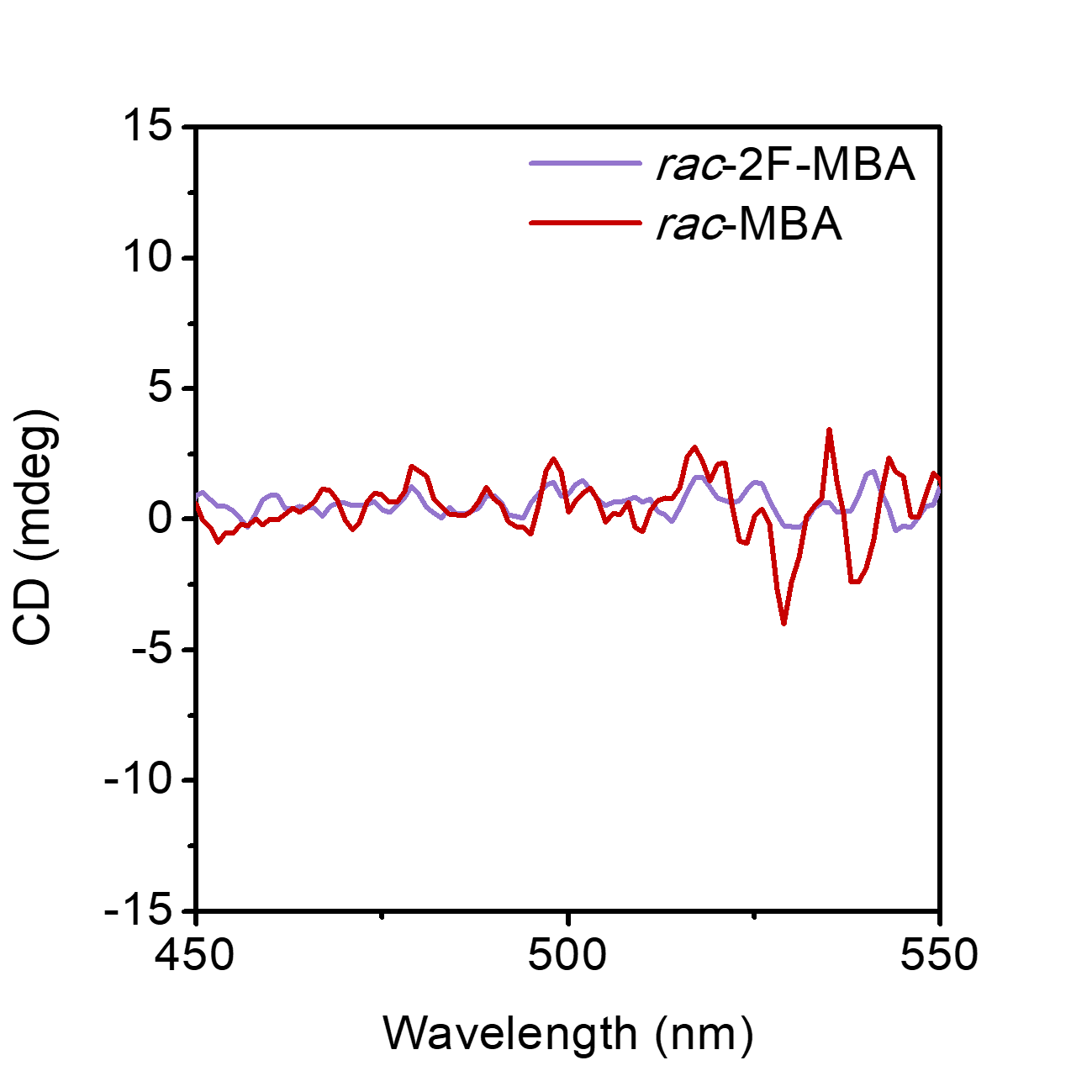
**

**Figure S8.** CD spectra obtained from the *rac*-OIHP thin film. Note that the *rac*-OIHP did not reveal CD spectra, indicating the absence of intrinsic chirality.


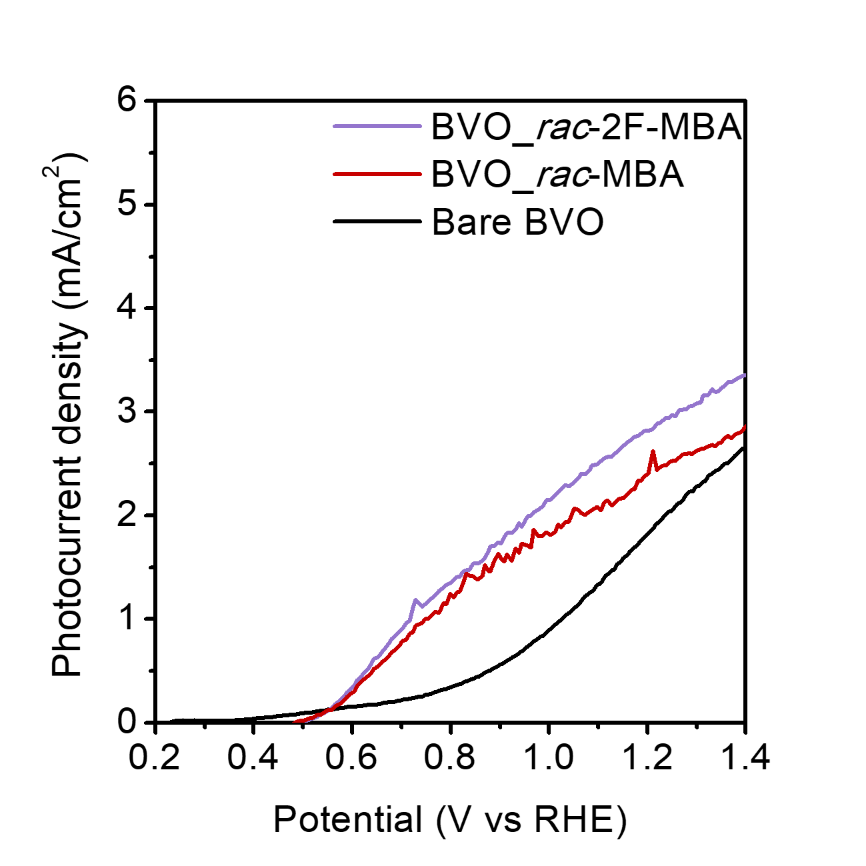


**Figure S9.** The LSV results measured from BVO_*rac*-OIHP devices. The device configuration is identical to one in Figure 2a, except for the replacement of the organic cation conformation from *S*- to *rac*- compound (*i.e*., FTO/SnO_2_/BVO/*rac*-OIHP/PTAA/NiFe/ NiFeOOH structure).

**
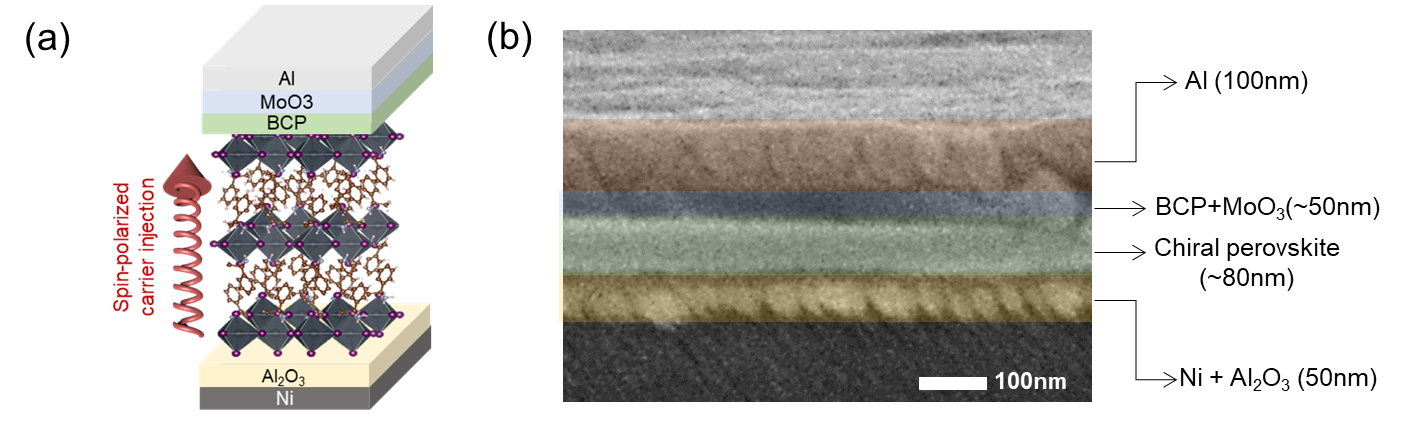
**

**Figure S10.** a) Schematic illustration of CISS measurement device.^[S2,S3]^ b) Cross-sectional SEM image of the CISS measurement device. The Ni electrode can polarize the spin state of the carriers in a specific direction depending on the magnetization direction. The Al_2_O_3_ (1.5 nm) plays the role as a spin tunneling buffer layer to reduce the conductivity mismatch between the Ni electrode and cOIHP thin films. The spin-polarized carriers from the Ni electrode tunnel through the helical potential of the repetitive chiral organic cations, being exposed to the augmented CISS effect. The bathocuproine (BCP) and molybdenum oxide (MoO_3_) prevent the injection of spin-unpolarized holes and electrons from the Al top electrode.

**
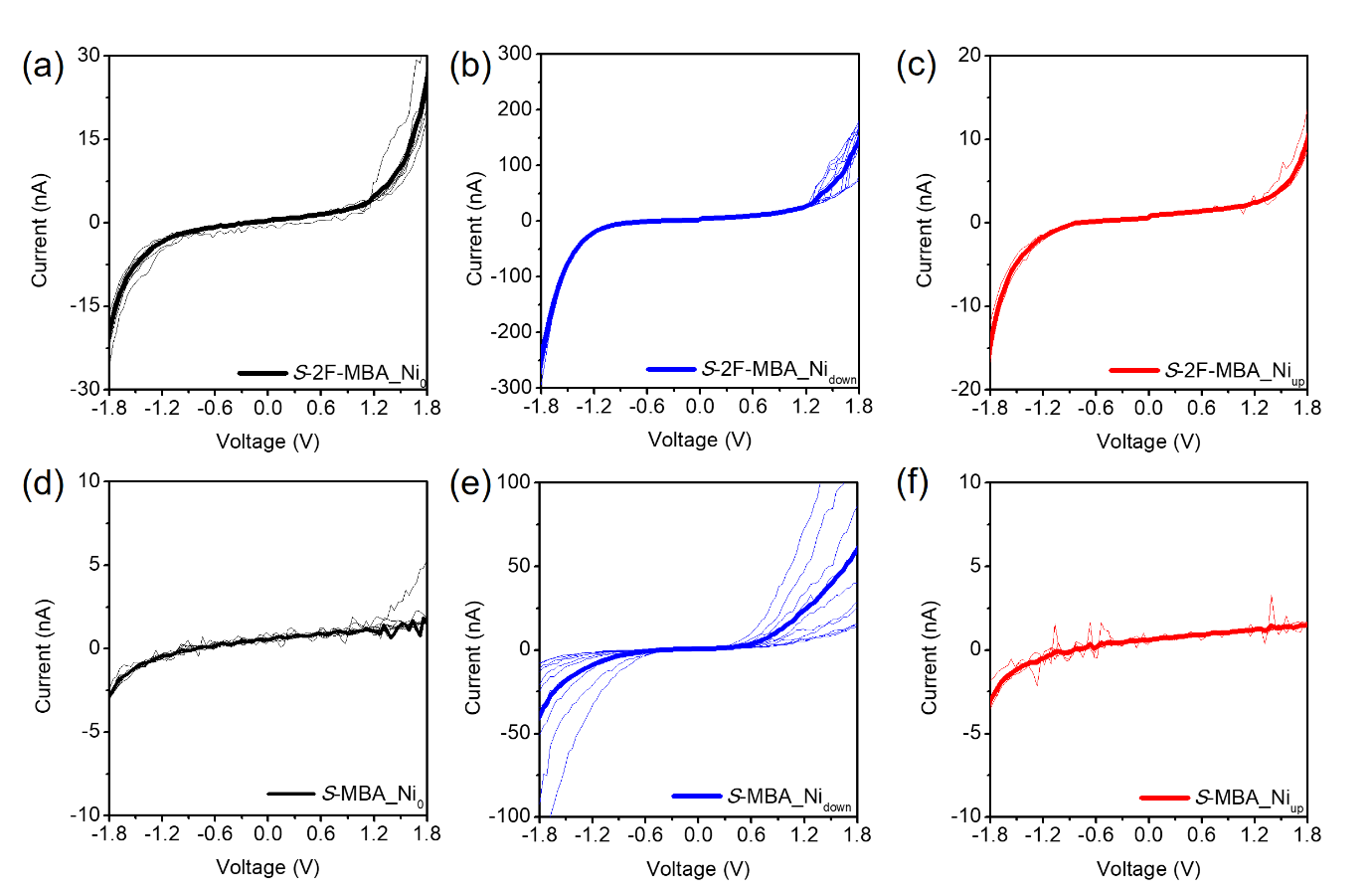
**

**Figure S11.** The black, blue, and red lines represent the various Ni magnetization directions of non-magnetization (Ni_0_), down (Ni_down_), and up (Ni_up_) direction, respectively. The bold lines indicate the mean values calculated from the 10 measurements: (a)-(c) for S-2F-MBA based cOIHP and (d)-(f) for S-MBA based cOIHP.

**
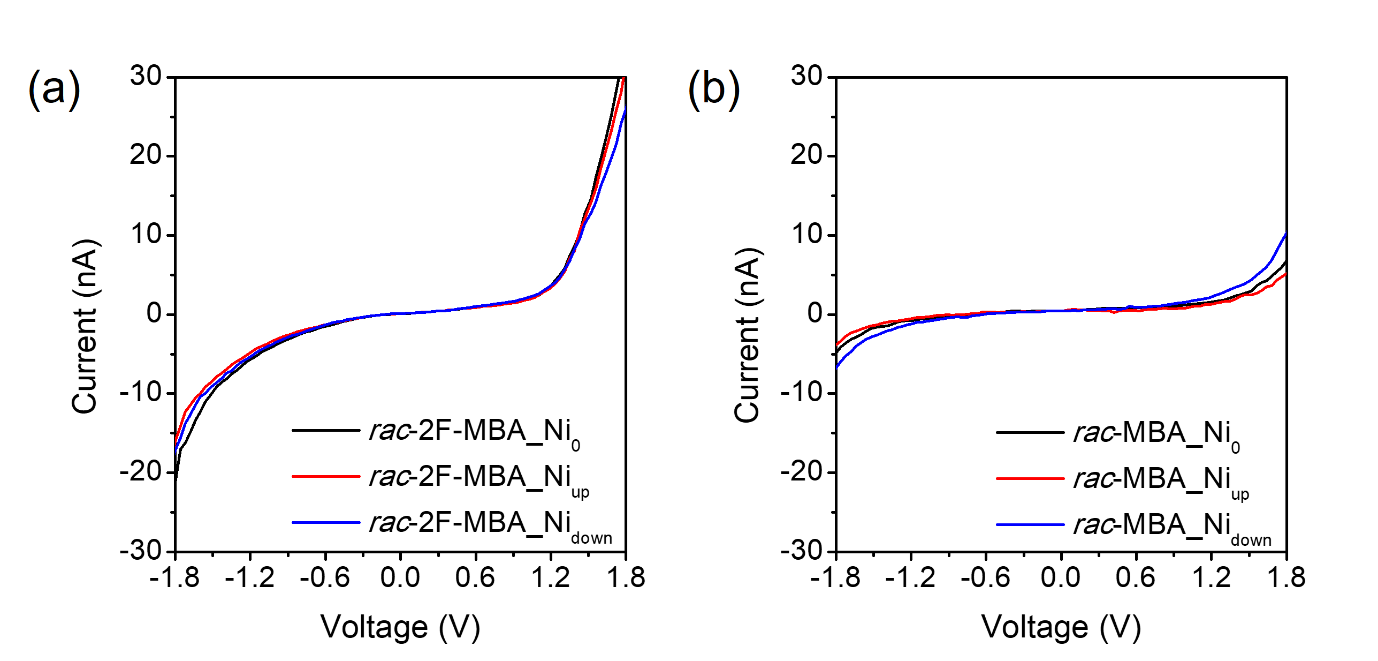
**

**Figure S12.** The I-V curves of the CISS measurement devices are averaged from 10 times measurements for each Ni magnetization direction for a) *rac*-2F-MBA OIHP and for b) *rac*-MBA OIHP.

**
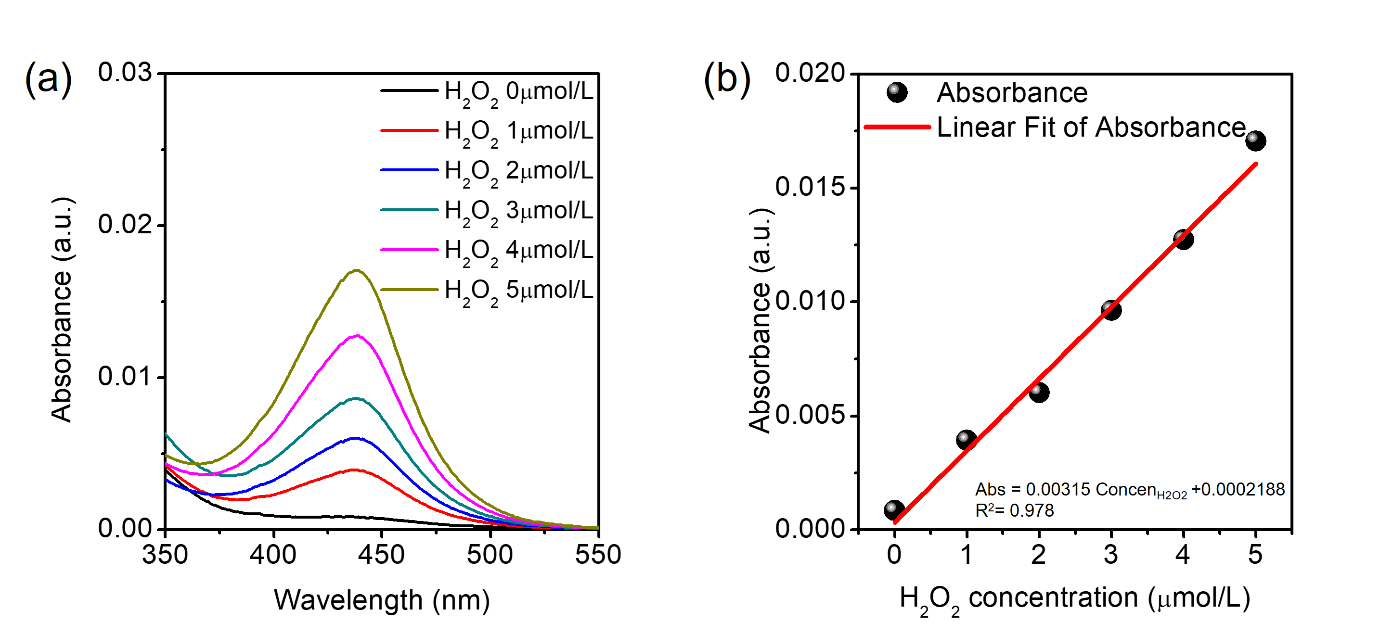
**

**Figure S13.** a) UV-vis absorption spectra by reacting 0.1 M Na_2_SO_4_ electrolyte with different concentrations of H_2_O_2_. The peak intensities were linearly increased as the H_2_O_2_ concentration increased. b) Linear fitting as a function of the H_2_O_2_ concentration for quantitively evaluation.

**
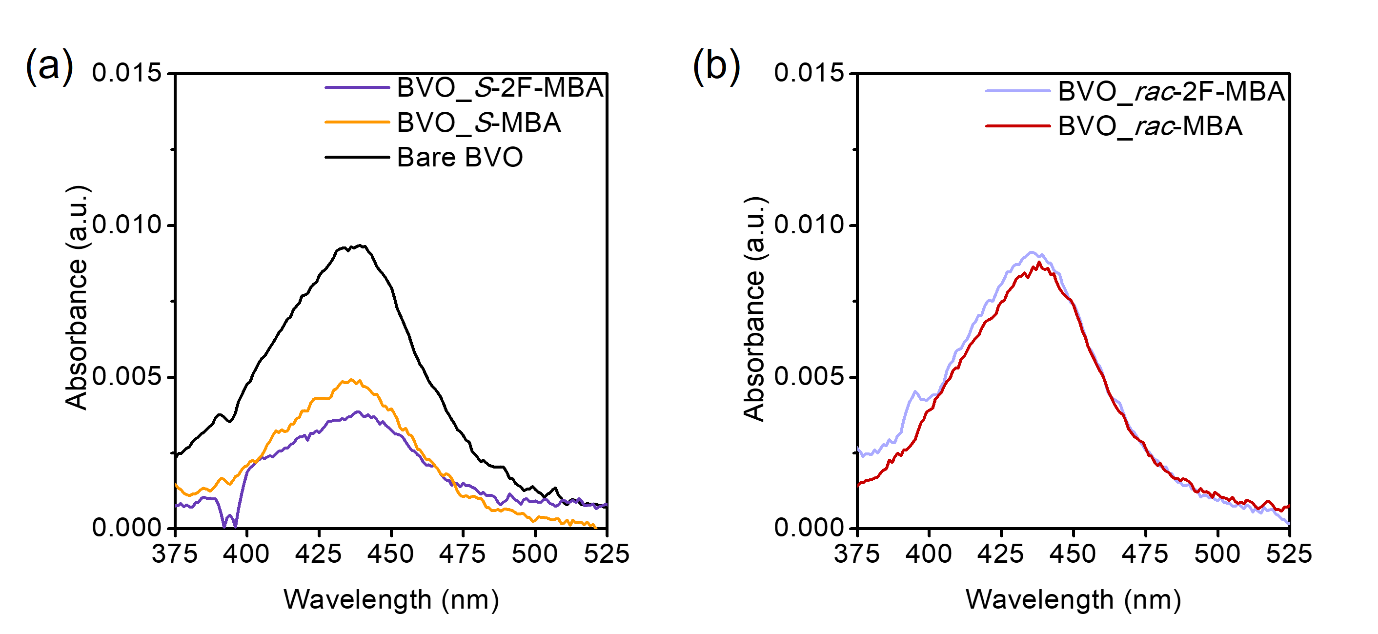
**

**Figure S14.** UV-vis absorption spectra for H_2_O_2_ detection using the *o*-tolidine-based colorimetric method in a 0.1 M Na_2_SO_4_ solution (pH 6.5). a) H_2_O_2_ detection for bare BVO and BVO_cOIHP devices. b) H_2_O_2_ detection for BVO_*rac*-OIHP device. All spectra were obtained after 60 min of oxygen evolution under 1-sun illumination.

**
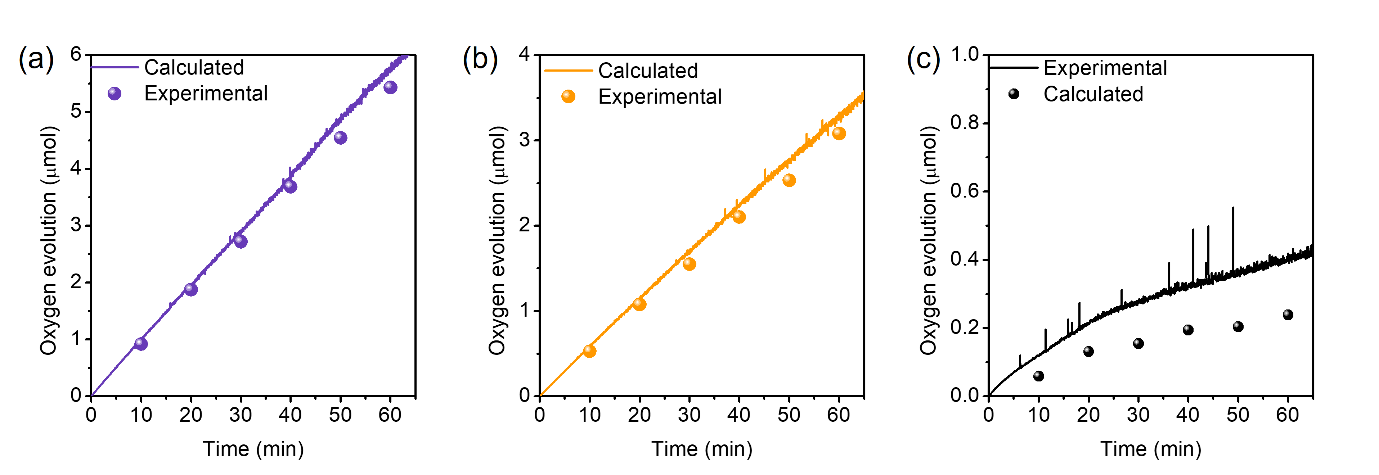
**

**Figure S15.** Oxygen evolution of the photoanode devices as a function of operation time. a) BVO_*S*-2F-MBA, b) BVO_*S*-MBA, and c) bare BVO photoanode devices.

**
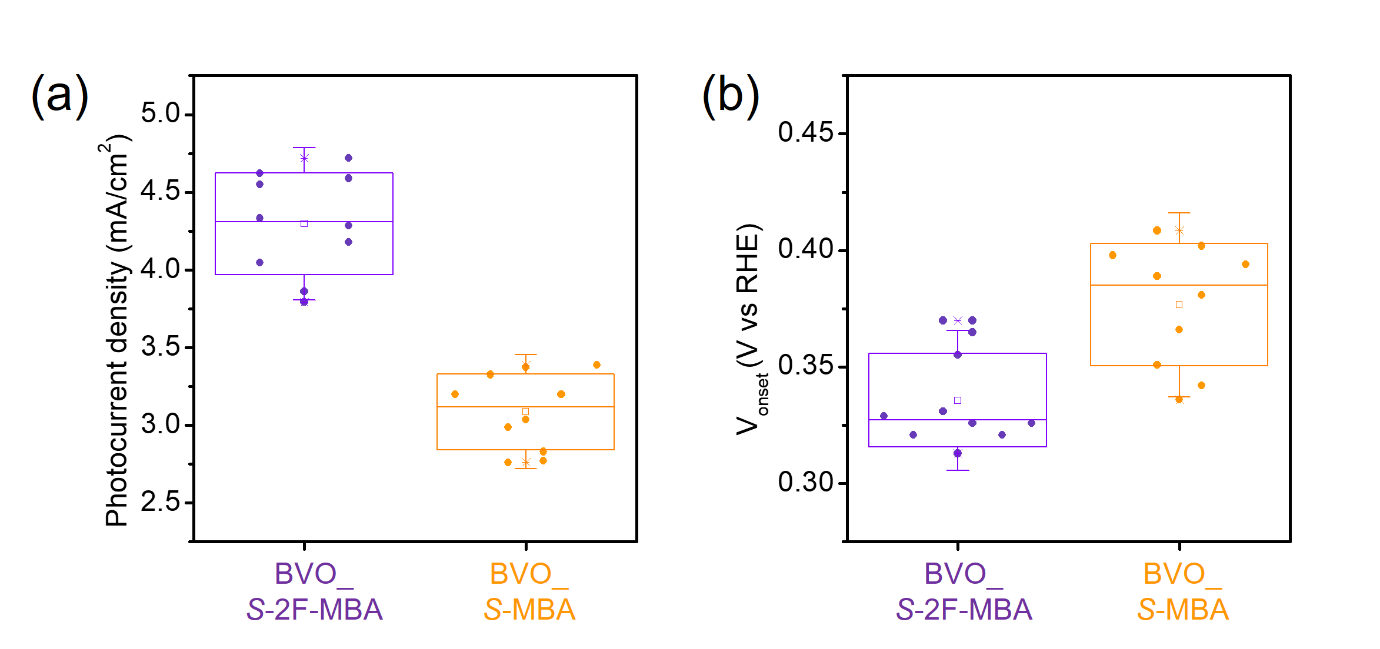
**

**Figure S16.** Statistical analyses obtained from the distinct 10 BVO_cOIHP photoanode devices. a) Photocurrent density and b) V_onset_ of the BVO_cOIHP devices. The plots were obtained from 10 different samples.

**
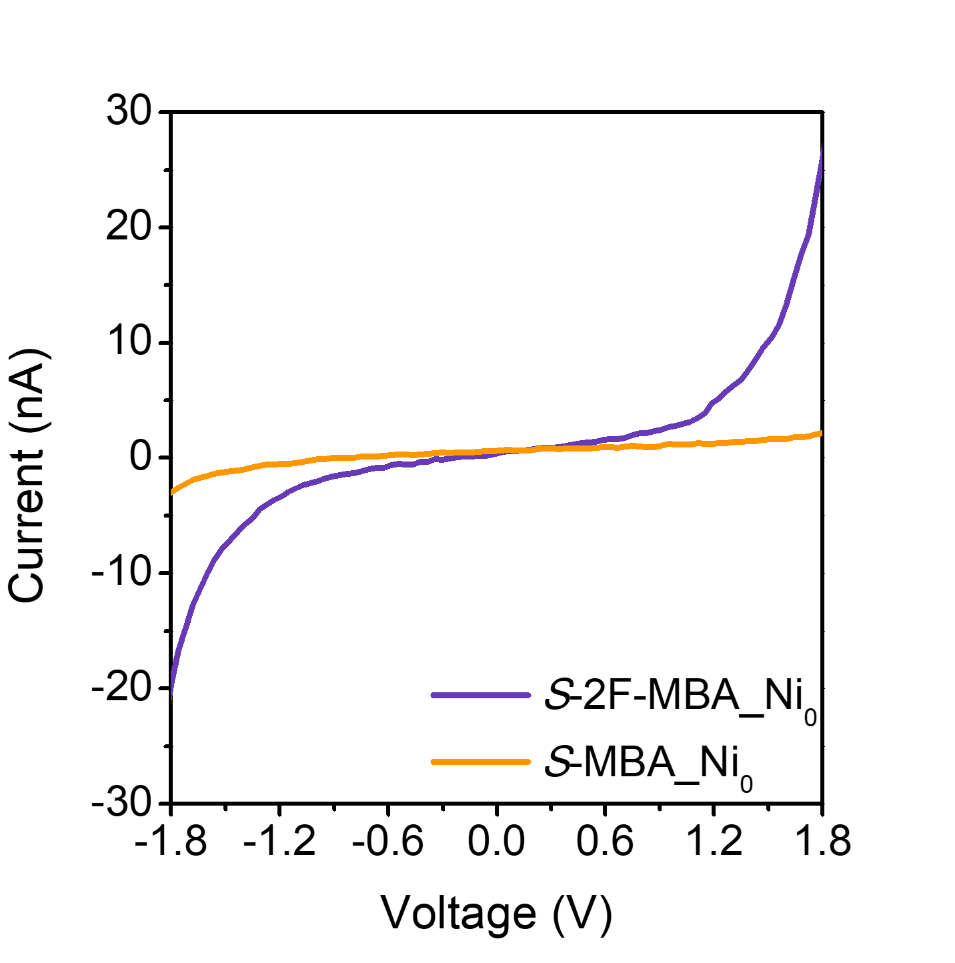
**

**Figure S17.** Comparison of the I-V curve when the Ni substrate in the CISS measurement device was not magnetized. The Ni­_0_ current represents the non-polarized carrier conductivity in the out-of-plane direction.

**
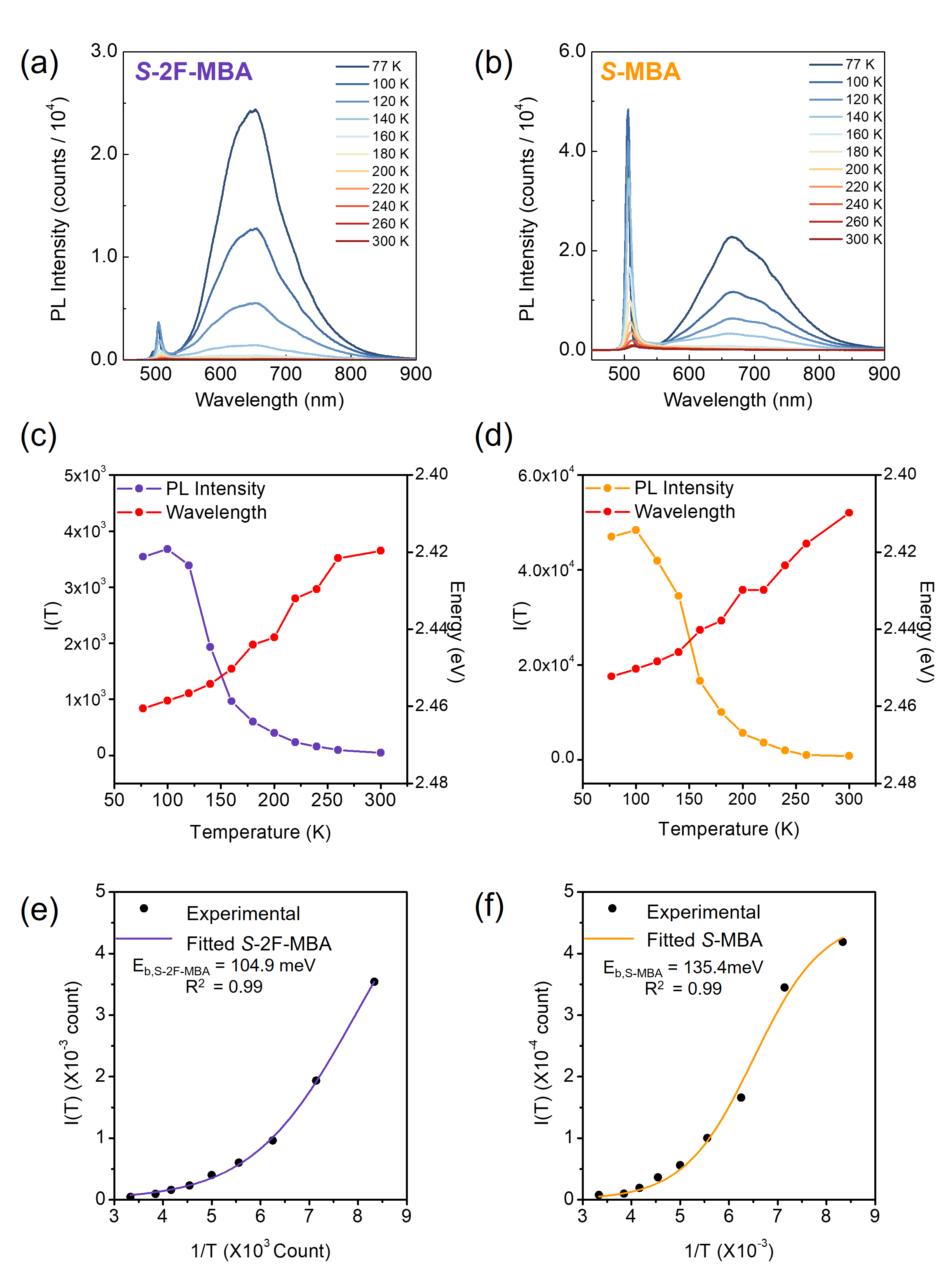
**

**Figure S18.** Temperature-dependent PL measurement excitation with a 375 nm laser source. The measurements were conducted on cOIHP thin films while increasing the temperature from 77 K to 300 K. The temperature-dependent PL spectra of a) *S*-2F-MBA cOIHP and b) *S*-MBA cOIHP. The plot of the temperature-dependent maximum PL intensity (I(T)) of the free excitonic emission and peak position of c) *S*-2F-MBA and d) *S*-MBA. The I(T) vs. inverse temperature plots for exciton binding energy extraction for e) *S*-2F-MBA cOIHP and f) *S*-MBA cOIHP.

**Supporting Note S1**

**Exciton binding energy (E_b_) extraction from temperature-dependent PL spectra**

The E_b_ for *S*-2F-MBA and *S*-MBA cOIHP was extracted from cryogenic to room-temperature PL analyses,^[S4]^ which were measured at 20 K intervals, as shown in Figure S18a and b. The free excitonic (FE) emission peak was marginally red-shifted with the increment of temperature. Also, the temperature-dependent intensity of the FE peaks (I(T)) was initially increased up to 100 K for *S*-2F-MBA and *S*-MBA OIHP. Sequentially, the I(T) was exponentially diminished along with the increased temperature (Figure S18c and d). Therefore, the E_b_ was calculated from the I(T) above 120 K for *S*-2F-MBA and *S*-MBA OIHP, following equation (S4) (Figure S16e and f):

$I\left( T \right)\text{= }\frac{I_{0}}{1 + B^{-E_{b}/\kappa_{B}T}}$ (S4)

where the I­_0_ indicates the initial intensity and the *κ*_B_ and B represent the Boltzmann constant and pre-exponential factor, respectively. The extracted E_b_ results are shown in Figure S16c and f. Furthermore, note that additional broad emission peaks assigned to the self-trapped exciton (STE) peaks were observed under 160 K (Figure S18a and b), regardless of the cation structures. However, the STE emission region was situated at a longer wavelength than the FE emission region, which is significantly separated from the FE region near the band edge. As a result, the STE did not interfere with the I(T).


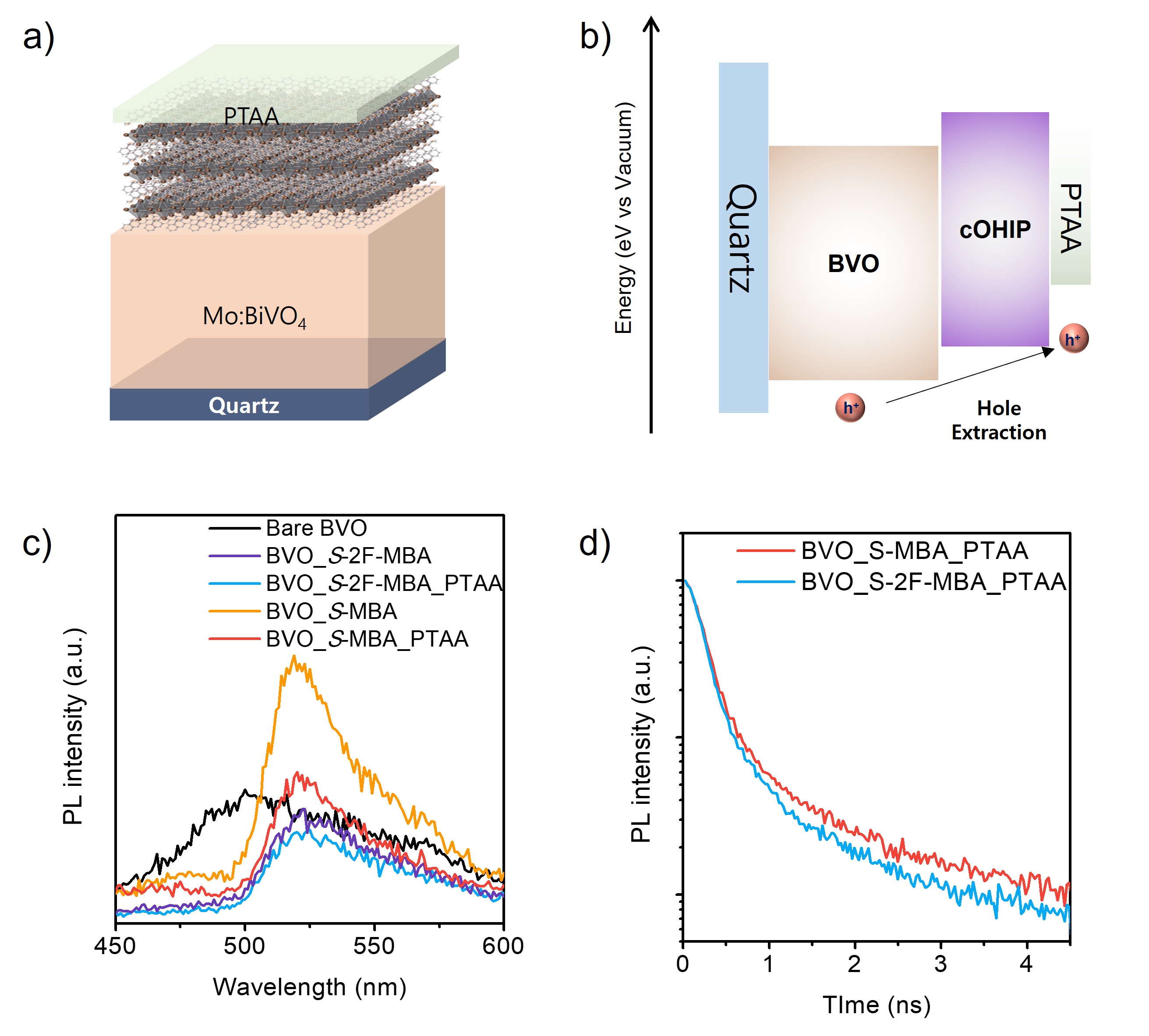


**Figure S19.** a) Schematic illustration of the HTL-only device with a configuration of quartz/BVO/cOIHP/PTAA. b) Schematic energy band diagram of the BVO_cOIHP_PTAA. c) The PL spectra measured under 370nm using the BVO_cOIHP (*i.e.*, without both HTL and ETL) and BVO_cOIHP_PTAA (*i.e.*, HTL-only device), respectively. d) TRPL spectroscopy of the HTL-only devices measured with an excitation wavelength of 370 nm.

**Table S2.** Result of bi-exponential fitting of TRPL decay curves and the deconvoluted parameters.

|  | **A_1_**  (%) | **τ_1_**  (ns) | **A_2_**  (%) | **τ_2_**  (ns) | **τ_avg_**  (ns) |
| --- | --- | --- | --- | --- | --- |
| **BVO_*S*-2F-MBA_PTAA** | 97.25298 | 0.21 | 2.747018 | 1.91 | 0.557471 |
| **BVO_*S*-MBA_PTAA** | 96.21211 | 0.24 | 3.78789 | 1.97 | 0.668787 |

**
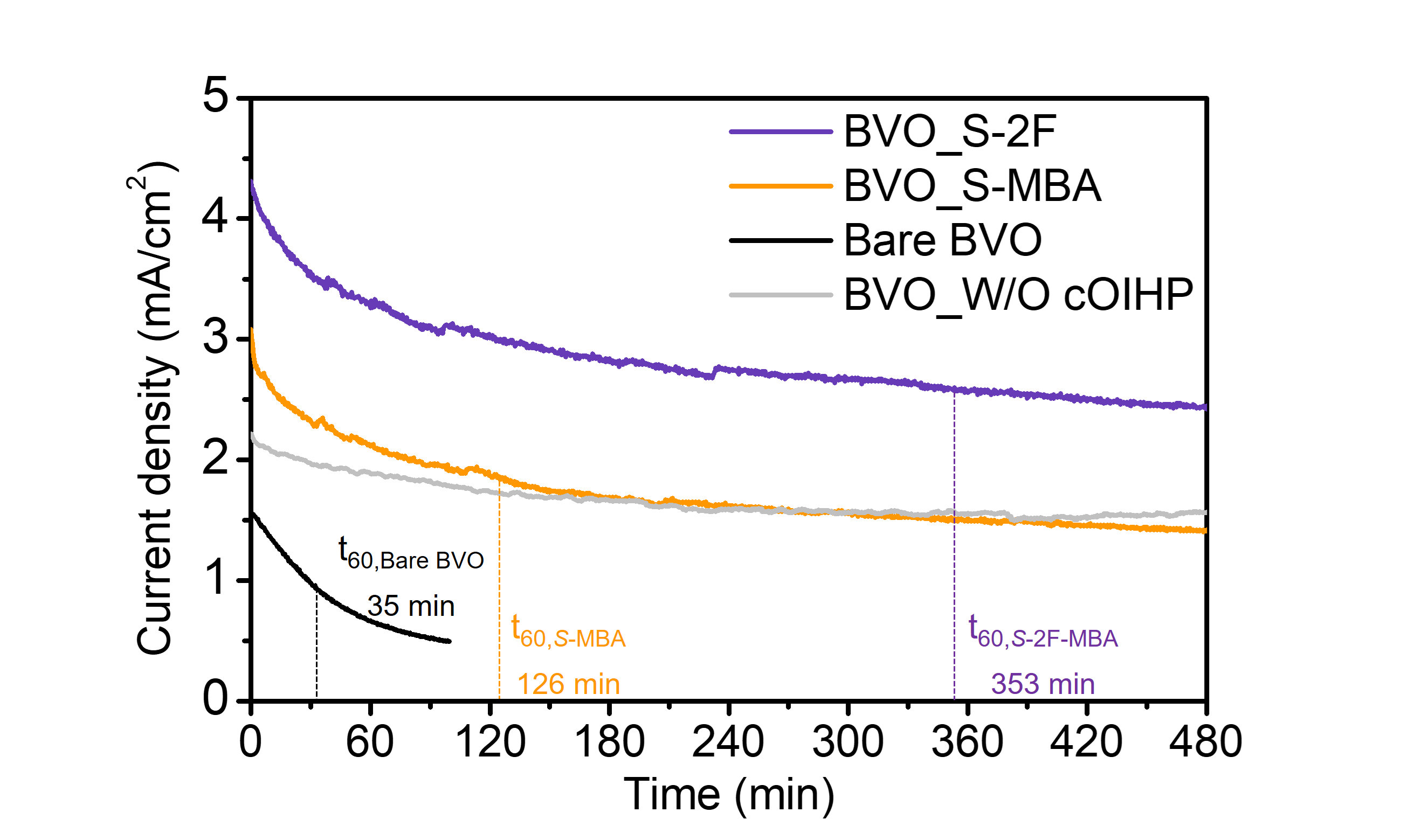
**

**Figure S20.** Operational stability of the BVO_cOIHP, bare BVO, and BVO_W/O cOIHP devices measured in a K-Bi electrolyte (pH 9) under 1 sun back illumination. The t_60_ indicates the time taken for photocurrent density to decrease by 60% from its initial value.

**
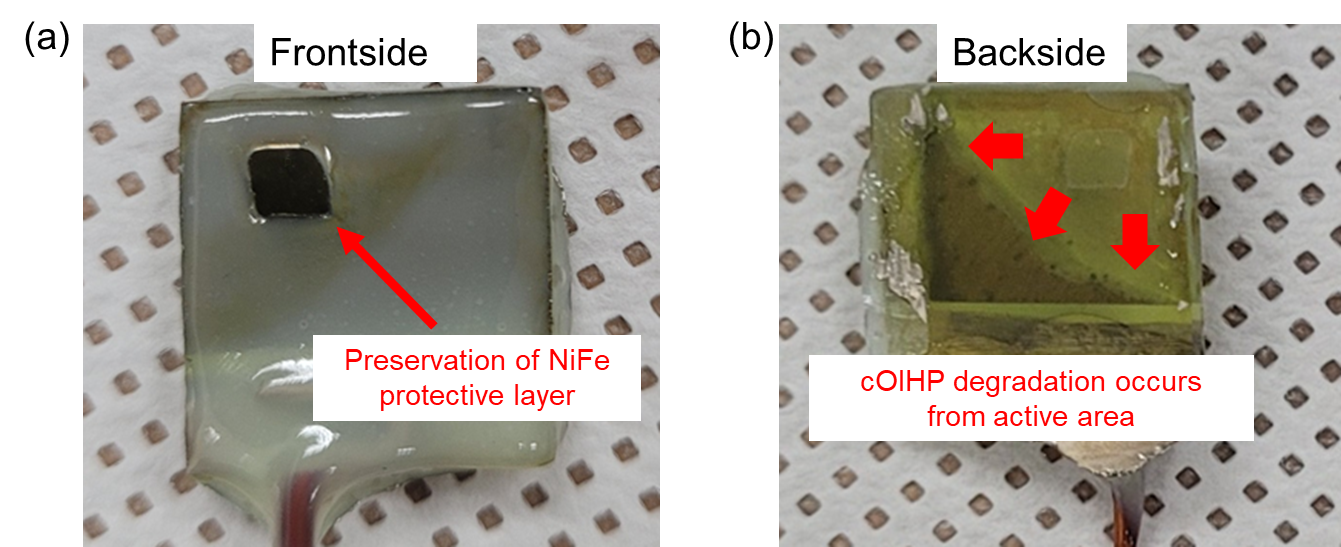
**

**Figure S21.** The spin-dependent OER photoanode devices after 24 h of operation in a K-Bi electrolyte (pH 9) under 1 sun illumination. Photographs of the a) frontside and b) backside.


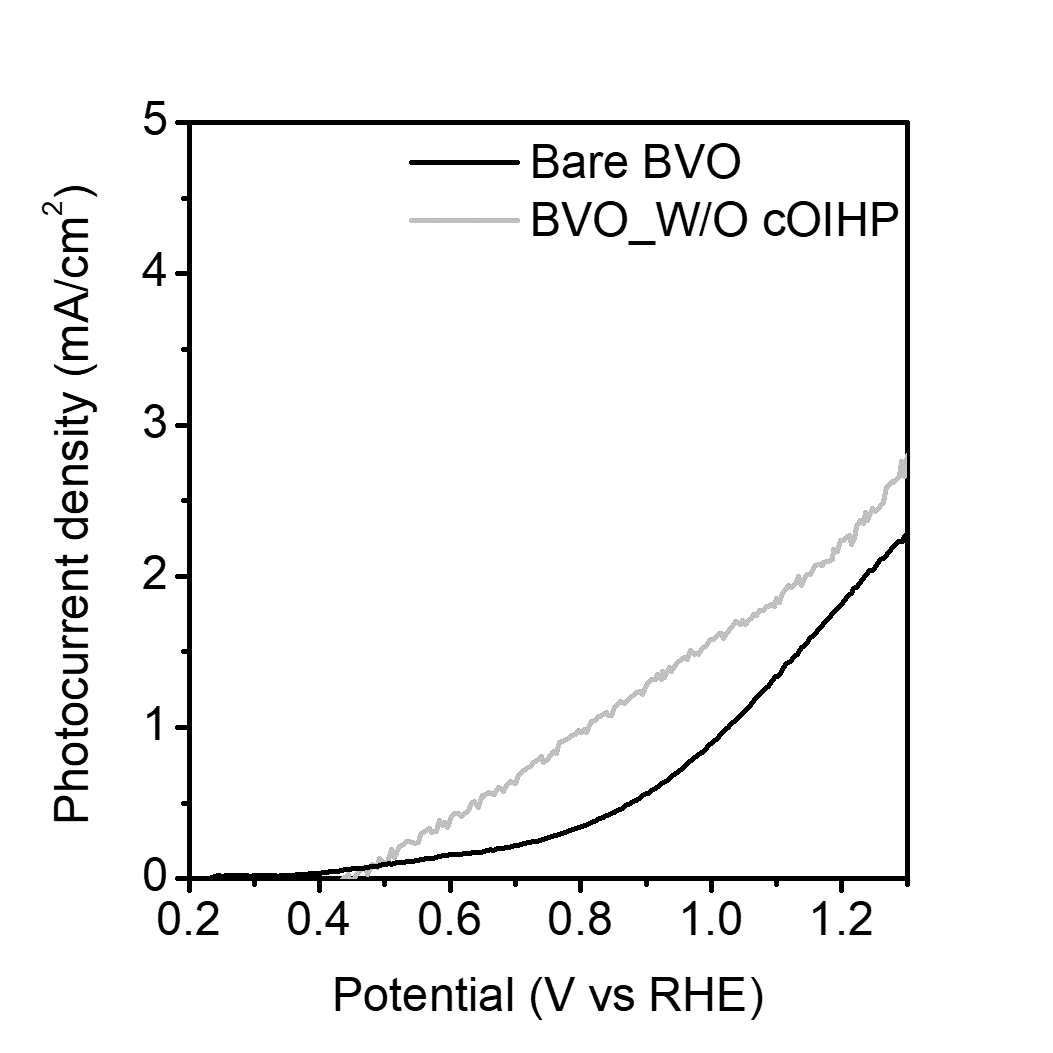


**Figure S22.** The LSV curve measured using the FTO/SnO2/BVO (*i.e*, bare BVO) and FTO/SnO2/BVO/PTAA/NiFe/NiFeOOH (*i.e.*, BVO_W/O cOHIP).

**
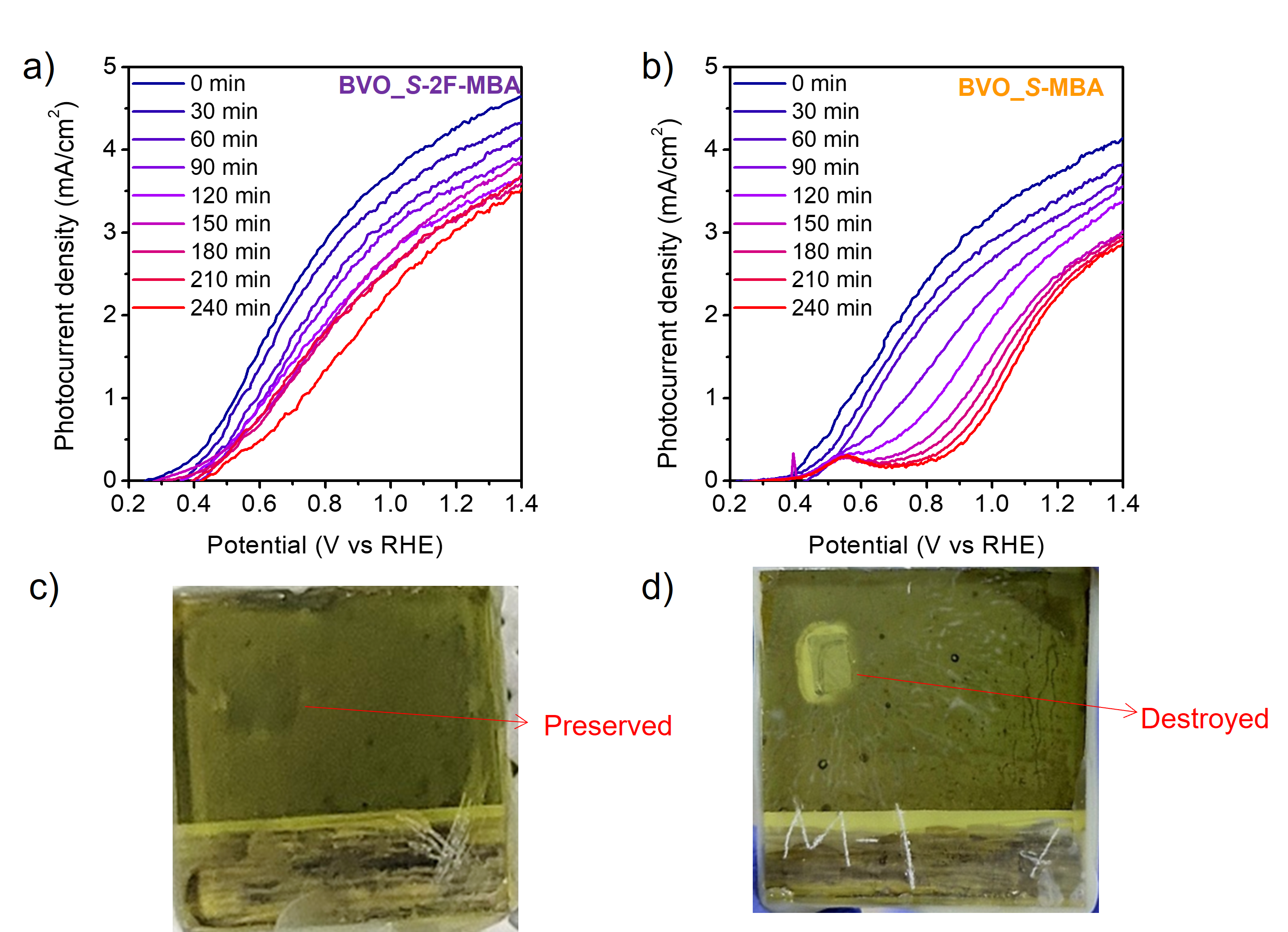
**

**Figure S23.** LSV curve recorded every 30 min during potentiostatic analysis in a K-Bi electrolyte (pH 9) under 1 sun back illumination for a) BVO_*S*-2F-MBA and b) BVO_*S*-MBA spin-dependent OER device. The digital photograph of the backside after 4 h operational stability test for c) BVO_*S*-2F-MBA and d) BVO_*S*-MBA cOIHP devices.

**
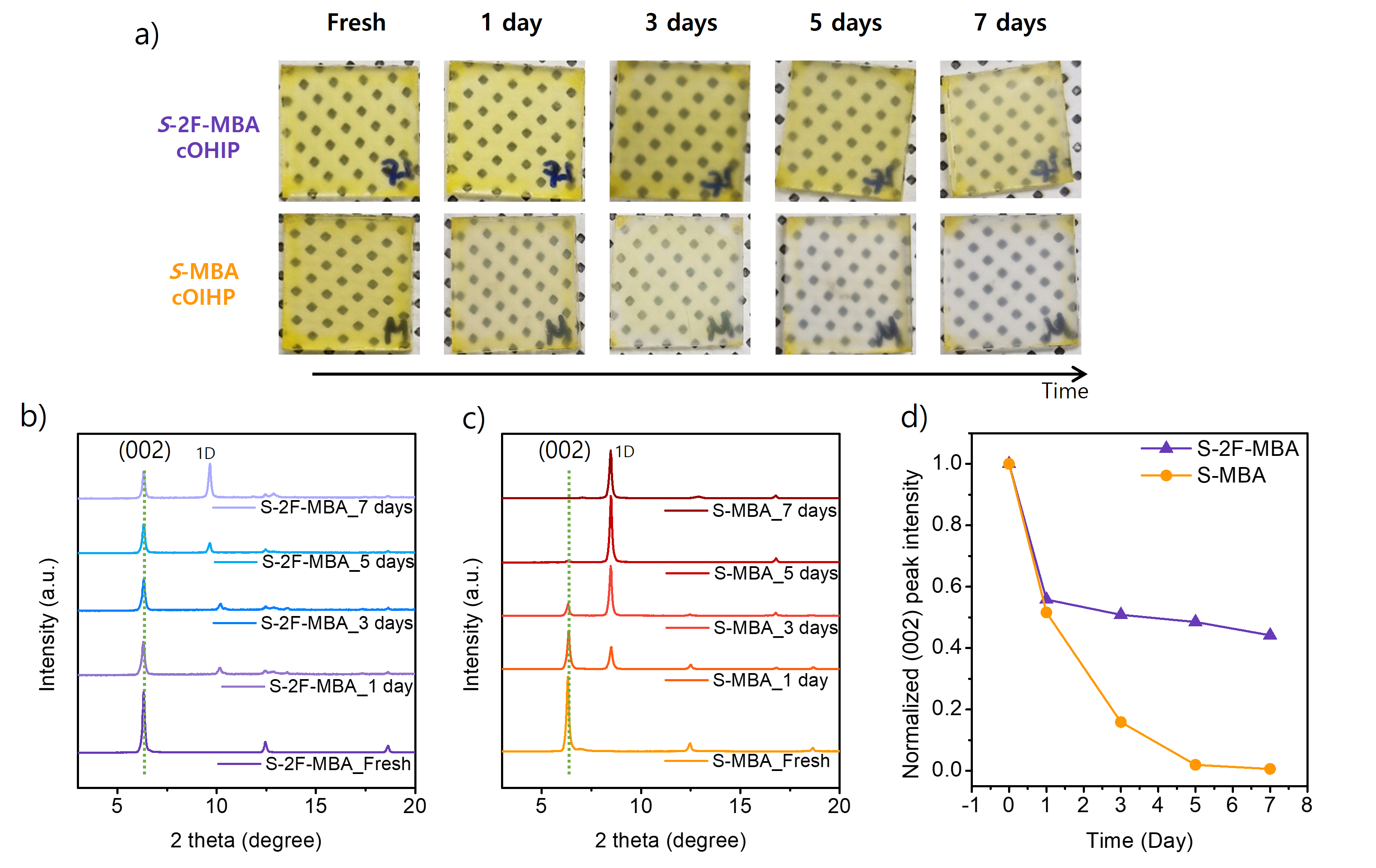
**

**Figure S24.** a) Digital photograph of the *S*-2F-MBA cOIHP (top) and *S*-MBA cOIHP thin films (down) viewed from the top surface during the humid test period under RH 85% at 25℃ using humidity-and temperature-controlled chamber (TH-PE-025, JEIO Tech, Korea). The humid stability of b) *S*-2F-MBA cOIHP and c) *S*-MBA cOIHP cOIHP was assessed by monitoring the XRD patterns for 7 days. The green dot lines indicate the (002) peaks at 6.32° for *S*-2F-MBA cOIHP and 6.34° for *S*-MBA cOIHP, respectively. d) Plot of the normalized (002) peak intensity obtained from (b) and (c), respectively.

**Supporting References**

[S1] J. Hu, I. W. H. Oswald, S. J. Stuard, M. M. Nahid, N. H. Zhou, O. F. Williams, Z. K. Guo, L. Yan, H. M. Hu, Z. Chen, X. Xiao, Y. Lin, Z. B. Yang, J. S. Huang, A. M. Moran, H. Ade, J. R. Neilson, W. You, *Nat. Commun.* **2019**, 10, 1276.

[S2] Y. H. Kim, Y. X. Zhai, H. P. Lu, X. Pan, C. X. Xiao, E. A. Gaulding, S. P. Harvey, J. J. Berry, Z. V. Vardeny, J. M. Luther, M. C. Beard, *Science* **2021**, 371, 1129–1133.

[S3] Y. H. Kim, R. Y. Song, J. Hao, Y. X. Zhai, L. Yan, T. Moot, A. F. Palmstrom, R. Brunecky, W. You, J. J. Berry, J. L. Blackburn, M. C. Beard, V. Blum, J. M. Luther, *Adv. Funct. Mater.* **2022**, 32, 2200454.

[S4] R. Chakraborty, A. Nag, *J. Phys. Chem. C.* **2020**, 124, 16177-16185.
